# A small molecule esculetin accelerates postprandial lipid clearance involving activation of C/EBPβ and CD36-mediated phagocytosis by adipose tissue macrophages

**DOI:** 10.7150/thno.110207

**Published:** 2025-04-28

**Authors:** Gang Wang, Zhaokai Li, Wei Ni, Heng Ye, Yang Liu, Linjian Chen, Lin Wang, Changjiang Liu, Jingyu Chen, Xuchao Wang, Xue Ding, Longshan Zhao, Xiaofeng Ge, Yan Wang, Yuanchao Ye, Toshihiko Kiwa, Linghe Zang, Jin Wang, Cuilian Dai, Binbin Liu

**Affiliations:** 1 Xiamen Cardiovascular Hospital, School of Medicine, Xiamen University, 2999 Jinshan Road, Xiamen, 361015, China.; 2 Shenyang Pharmaceutical University, 103 Wenhua Road, Shenyang, 110016, China.; 3 Interdisciplinary Science and Engineering in Health Systems, Institute of Academic and Research, Okayama University, Okayama, 700-8530, Japan.; 4 Department of Medicine, University of Washington, 750 Republican Street, Seattle, WA, 98109, USA.

**Keywords:** adipose tissue macrophage, CD36, C/EBPβ, esculetin, high-density lipoprotein

## Abstract

**Rationale**: Adipose tissue buffers dietary lipids to maintain postprandial lipid homeostasis. Adipose tissue macrophages (ATMs) mediate the phagocytosis of postprandial lipids from the exogenous diet, generating high-density lipoprotein (HDL) particles that facilitate lipid circulation and excretion. However, the underlying mechanisms remain poorly understood. This study investigates the effects of esculetin, a coumarin compound, on postprandial cholesterol circulation and excretion following a high-fat meal.

**Methods**: Mice were fed a lipid-rich meal for three days to assess the effects of esculetin on postprandial lipid circulation, using serum lipid profiling and metabolomics analysis. Epididymal white adipose tissue (eWAT) removal and flow cytometry were performed to analyze ATMs and confirm their role in mediating esculetin's effects on postprandial lipemia. Epigenetic profiling, transcriptome analysis, chromatin immunoprecipitation, and Terahertz chemical microscopy were employed to elucidate the molecular targets and mechanisms of esculetin.

**Results**: Esculetin significantly elevates postprandial HDL cholesterol levels to values comparable to pitavastatin and modifies serum metabolites involved in bile-mediated cholesterol excretion, leading to increased bile acid concentrations in the bile. This effect is mediated by an increased ratio and phagocytic activity of a subset of ATMs expressing the scavenger receptor CD36, as eWAT removal and CD36 blockade inhibit this response. Furthermore, esculetin enhances the uptake of oxidized LDL via CD36, as demonstrated in cultured macrophages, and induces epigenetic changes controlled by the key transcription factor C/EBPβ, accompanied by increased C/EBPβ binding to the *Cd36* promoter. A direct interaction between esculetin and C/EBPβ was observed using Terahertz chemical microscopy. Additionally, the activation of C/EBPβ by esculetin in ATMs was confirmed *in vivo*.

**Conclusion**: Esculetin accelerates postprandial lipid circulation by binding to C/EBPβ and enhancing CD36-dependent phagocytosis in ATMs.

## Introduction

After the consumption of a lipid-rich meal, dietary fats undergo digestion, absorption, and packaging into chylomicrons in the small intestine. Chylomicrons, which are rich in triglycerides and cholesterol, are transported from the intestinal villi via the lymphatic system to the venous circulation, bypassing the portal circulation 1. Adipose tissue constitutes the primary site for chylomicron clearance, wherein lipoprotein lipase (LPL) abundantly expressed in adipocytes hydrolyzes chylomicrons 2. This process generates chylomicron remnants and directs dietary cholesterol toward three metabolic fates: hepatic utilization for lipoprotein synthesis, biliary excretion via reverse cholesterol transport, or intracellular storage within lipid droplets 3,4. Notably, adipose tissue maintains a net importing state of dietary lipids for approximately 5 hours postprandially and throughout most daytime hours under a typical three-meal dietary pattern. The tissue promptly responds to circulating chylomicrons and chylomicrons remnants via LPL-mediated hydrolysis, thereby mitigating lipid retention in the bloodstream 5. This metabolic coordination extends to cross-talk with hepatic and muscular systems that collectively determine lipid partitioning and clearance pathways 6. Visceral adipose tissue is more metabolically active than subcutaneous adipose tissue and can exert a greater influence on lipid metabolism 7, particularly in the context of postprandial lipid handling. This clearance mechanism of postprandial lipid critically determines postprandial lipid levels, with impaired adipose tissue function leading to prolonged circulation of chylomicrons remnants that may exacerbate atherosclerosis risk and insulin resistance 8,9. Thus, adipose tissue acts as a buffer for daily lipid flux 10, although the underlying mechanisms remain largely unknown.

Macrophages in liver and adipose tissue, monocytes in blood or others take up and hydrolyze excess cholesterol into free cholesterol, which undergoes macrophage cholesterol efflux (MCE) out of the cells via ATP binding cassette transporters or spontaneous diffusion 11. HDL cholesterol generated in this manner facilitate reverse cholesterol transport (RCT), carrying cholesterol from peripheral tissues to the liver 12,13. There, cholesterol is converted into bile acids, an essential step in bile-mediated cholesterol excretion. Elevated MCE and HDL-driven RCT promotes the postprandial cholesterol circulation and clearance and is therefore negatively associated with atherosclerotic cardiovascular disease risk 14,15. In postprandial cholesterol metabolism, emerging evidence highlights the pivotal involvement of adipose tissue macrophages (ATMs), particularly a specialized subpopulation expressing Tim4, CD36, and ABCA1 16,17. Depletion of these macrophages has been shown to reduce postprandial increases in HDL cholesterol levels and liver Kupffer cells and peritoneal cavity macrophages play minor roles in postprandial HDL cholesterol metabolism 18.

The class B scavenger receptor CD36-mediated lipid uptake pathway demonstrates dual regulatory effects: under physiological conditions, it supports lipid homeostasis through chylomicrons remnant recognition and phagocytosis, whereas pathological hyperactivation in obesogenic environments may induce cholesterol overload and foam cell formation, exacerbating metabolic dysregulation 19,20. This highlights the critical balance that CD36 must maintain between lipid clearance and metabolic stability during postprandial states. In Japanese patients with type I CD36 deficiency, oral fat loading induces postprandial hyperlipidemia and the accumulation of intestinal-derived lipids 21,22. This deficiency similarly compromises the clearance of dietary lipids following high-fat meal consumption, suggesting CD36's essential role in postprandial lipid processing 23. Thus, CD36-mediated phagocytosis, especially in ATMs is essential for the clearance of postprandial lipids. Moreover, therapeutic strategies targeting CD36-mediated phagocytosis to modulate postprandial HDL cholesterol are lacking.

Current therapeutic strategies, notably HMG-CoA reductase inhibitors (statins), primarily target hepatic cholesterol synthesis but exhibit limited efficacy in modulating adipose tissue-mediated postprandial lipid metabolism. Some statins can also increase HDL levels, which theoretically play a minor role in regulating postprandial cholesterol metabolism. And, accumulating evidence suggests that statin therapy may offer limited therapeutic efficacy for dyslipidemia and could lead to potential side effects, even with short-term use. These side effects include myalgia, rhabdomyolysis, new-onset diabetes mellitus, cognitive impairment, and hemorrhagic stroke 24,25. Given the central role of adipose tissue in meal-derived lipid clearance, developing adipose-centric therapeutic approaches presents promising avenues for managing postprandial hyperlipidemia. Therefore, exploring compounds with alternative mechanisms is critical. Previous investigations suggest esculetin may modulate adipose tissue metabolism potentially through ATM-mediated lipid handling and cholesterol clearance pathways. Esculetin (Figure S1A), a coumarin compound enriched in various plants including* Sonchus grandifolius*, *Aesculus turbinate* and* Cortex Fraxinis*, has been reported to exert anti-inflammatory effects, involving in inhibiting MAPK activation, AP-1 and NF-κB activation, and PI3-kinase activation via Ras 26,27. Esculetin also demonstrates anti-obesity effects by activating AMP-activated protein kinase and suppressing PPARγ expression in adipose tissues 28, and regulates serum lipid profiles and shows potential anti-atherosclerosis benefit 29,30. These findings prompted our investigation into its potential role in regulating postprandial cholesterol metabolism.

In this study, mice were fed a lipid-rich meal for only 3 days to investigate the effects of esculetin on postprandial lipemia and lipid metabolism induced by the meal. The metabolic changes induced by esculetin were analyzed using ultra-high-performance liquid chromatography-tandem mass spectrometry (UHPLC-MS/MS). Visceral adipose tissue removal surgery was employed to confirm the role of visceral adipose tissue in the effect of esculetin on postprandial lipemia. Flow cytometry was used to assess esculetin's impact on ATM-mediated lipid uptake and CD36-mediated phagocytosis by ATMs, elucidating the mechanism through which esculetin regulates postprandial lipemia. Chromatin accessibility profiling via assay for transposase-accessible chromatin using sequencing (ATAC-seq) and transcriptome analysis through RNA sequencing identified CCAAT enhancer-binding protein beta (C/EBPβ) as a crucial transcription factor. The interaction between esculetin and C/EBPβ was confirmed using terahertz chemical microscopy. In summary, esculetin was found to increase postprandial HDL cholesterol levels and enhance CD36-mediated phagocytosis by ATMs, implicating C/EBPβ in these effects.

## Methods

### Drug and reagents

Esculetin (Cat No.: D77970, Lot No.: D40036020, ACMEC, Shanghai, China) was dissolved in 2.5% Tween-80 for mice treatment by oral gavage or dimethyl sulfoxide for cell treatment. Simvastatin (Approval number: H20084420, Lot No.: 20221018, XinQi Pharmaceutical Co. LTD, Shandong, China) and pitavastatin (Approval number: H20193061, Lot No.: B221070, Salubris Pharmaceutical Co. LTD, Shenzhen, China) were dissolved in ddH_2_O for mice treatment by oral gavage. The reagents used in this work are listed in Table S1.

### Mice and treatment

C57BL6J male mice (8-9 weeks old) were housed in specific pathogen free facilities with 12 h light:12 h dark cycles at 22-23 °C in Xiamen University. All experiments were conducted in accordance with the NIH guidelines for the Care and Use Laboratory Animal and approved by the Animal Care and Protection Committee of Xiamen University (Protocol No. XMULAC20200150).

**Figure 1:**
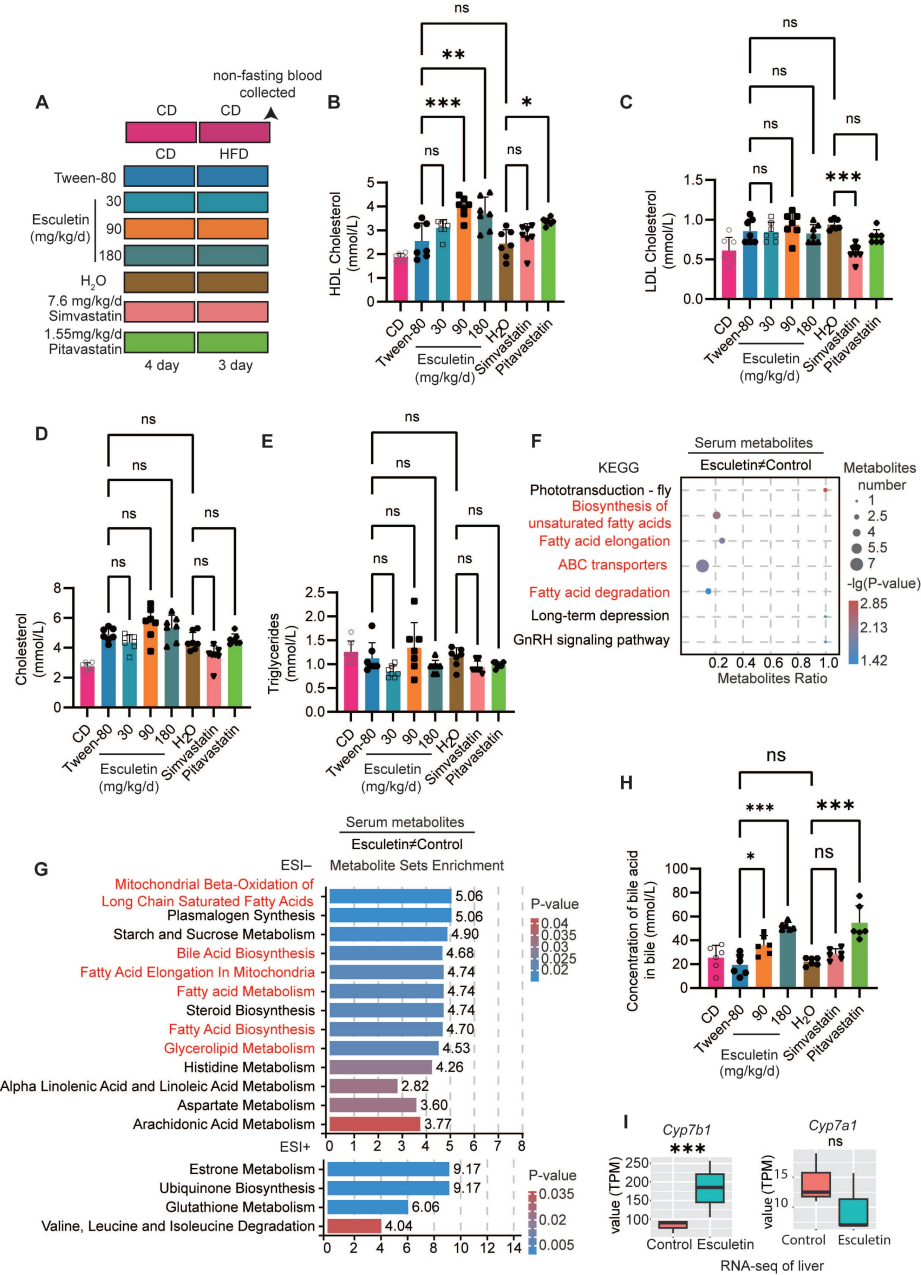
** Esculetin increases postprandial HDL cholesterol levels and enhances HDL-mediated cholesterol excretion. (A)** Schematic representation of the experimental design, including mouse treatments and sample collection procedures. Mice were administered esculetin, simvastatin, pitavastatin, or a vehicle control (Tween-80 or H_2_O) via oral gavage for 7 days, alongside 3 days of high-fat diet feeding. Negative control mice were fed a control diet. **(B-E)** Quantification of postprandial serum lipids. Data points represent individual mice (n = 7) across two independent experiments, analyzed using one-way ANOVA with Tukey's multiple comparisons test. **(F)** Kyoto Encyclopedia of Genes and Genomes (KEGG) enrichment analysis was performed on esculetin-induced significantly differentially expressed metabolites (DEMs) (n = 6). **(G)** Metabolite set enrichment analysis. **(H)** Bile acid levels in bile were quantified. Data points represent individual mice (n = 6) across two independent experiments, analyzed using one-way ANOVA with Tukey's multiple comparisons test. **(I)** Transcription levels of *Cyp7a1* and *Cyp7b1*, expressed as transcripts per kilobase million (TPM), were quantified by RNA-seq analysis of liver samples from high-fat diet-fed mice treated with a control or 90 mg/kg/day esculetin (n = 3).

Considering that the doses of esculetin used *in vivo* reportedly ranged from 10 31 to 200 mg/kg/d 32, a series of doses of esculetin (0, 30, 90, 180 mg/kg/d) were daily administered to the mice by oral gavage for 7 days. A high dose of simvastatin (7.6 mg/kg/d, equivalent to 60 mg/d for humans) or pitavastatin (1.55 mg/kg/d, equivalent to 12 mg/d for humans) was daily administered by oral gavage for 7 days as positive controls. In the first 4 days, the mice were fed with a control diet (CD) containing 10% of kcal from fat. Then, the mice were fed with a high-fat diet (HFD) containing 60% of kcal from fat for an additional 3 days to understand lipid-rich meals-induced postprandial lipid circulation, in accordance with previous reports 18,33. The control group was fed with CD for 7 days (Figure 1A). Furthermore, 40 mg/kg/d Sulfo-N-succinimidyl oleate (SSO) was daily administered to mice from Day 4 to 7.

### Flow cytometry

The enzymatic digestion of eWAT or liver was performed using 1 mg/mL collagenase D or 0.1 mg/mL Liberase, following the procedure outlined in our previous report 33. This enzymatic digestion was carried out in RPMI 1640 containing 1% fetal bovine serum and 1 μg/mL DNase I for 35 min at 37 °C. Subsequently, Percoll density gradient separation (70%-40%) was employed to enrich the macrophage population. After FcR blocking, cells were stained with fixable viability dye and fluorescently conjugated antibodies in MACS buffer (1× phosphate-buffered saline containing 1% BSA and 2 mM EDTA) for 30 min on ice.

For lipid staining, cells were fixed in IC Fixation Buffer and stained with LipidTox (1:200 dilution) for 30 min at room temperature. To stain C/EBPβ, cells were fixed and permeabilized using the eBioscience Foxp3/transcription factor staining buffer set after staining with cell surface proteins. The anti-C/EBPβ antibody (1:1000 dilution) was incubated overnight at 4 °C, followed by staining with Alexa Fluor 488-labeled secondary antibody (1:800 dilution) for 2 h on ice.

Samples were processed using the FACS LSR Fortessa system (Becton Dickinson, Franklin Lakes, USA), and the data were analyzed using Flow Jo software. ATMs were collected using a BD FACSAria III cell sorter (Becton Dickinson, Franklin Lakes, USA), and the cells were sorted into a 48-well plate with complement culture medium. Doublet or multiplet cells were excluded, and non-viable cells were excluded by gating on fixable viability dye-negative cells. The representative gating strategies of flow cytometry analysis are referred to in Figure S4B. The antibodies used in this work are listed in Table S1.

### Surgical removal of eWAT

Male 9-week-old mice were divided into two groups: a sham group and a surgery group. First, all mice were anesthetized with 2% isoflurane during the procedure. In the eWAT surgical removal group, both eWAT fat pads were surgically removed through a mid-ventral abdominal incision without damaging the testicular blood supply 34. In the sham group, a mid-ventral incision was made, and the epididymal fat pads were visualized, pulled out, but left intact and placed back inside the peritoneal cavity. One week after surgery, the mice were administered 0 or 90 mg/kg/d of esculetin for 7 days, including 3 days on a high-fat diet as described above.

### Cell culture

RAW 264.7 cells, a mouse macrophage cell line, and sorted ATMs were cultured in Dulbecco's modified Eagle's medium (DMEM) supplemented with 10% fetal bovine serum, 100 U/ml penicillin and 0.1 mg/ml streptomycin at 37 °C under 5% CO_2_.

RAW 264.6 cells were transfected with the LentiCRISPR v2 plasmid containing gRNA against *Cebpb* or empty vector using the Gene Pulser Xcell Eukaryotic System (165-2661, Bio-Rad, CA, USA) 35 and selected by puromycin for 3 days. The gRNA sequence against *Cebpb* is listed in Table S1. A concentration of 25 μg/mL DIL-ox-LDL, esculetin, or SSO, as indicated, was added to RAW 264.7 cells for the specified time periods.

### Lipid profile analysis and hematology analysis

Serum and bile samples were obtained from non-fasted mice, and the concentrations of postprandial triglycerides, HDL, LDL, total cholesterol, alanine aminotransferase, aspartate transaminase, and bile acid were quantified at the Clinical Laboratory of Xiamen Cardiovascular Hospital.

### Metabolomics study

The serum samples from the mice were combined with an isotopically labeled internal standard mixture and an extraction solution comprising 50% methanol and 50% acetonitrile. Following sonication and protein precipitation, the resulting supernatants were employed for UHPLC-MS/MS analysis 33.

A UHPLC system (Vanquish, Thermo Fisher Scientific) equipped with an ACQUITY UPLC BEH Amide column (2.1 mm × 50 mm, 1.7 μm, Waters Corp., MA, USA) coupled to an Orbitrap Exploris 120 mass spectrometer (Orbitrap MS, Thermo Fisher Scientific) was employed for UHPLC-MS/MS analysis. The mobile phase system consisted of buffer A (acetonitrile) and buffer B, composed of 25 mM ammonium ethanoate and 25 mM ammonium hydroxide. The UHPLC elution program was as follows: 0-11 min, 15% buffer A; 11-12 min, 75% buffer A; 12-14 min, 98% buffer A; 14-14.1 min, reduced to 15% buffer A; 14.1-16 min, 15% buffer A. The flow rate was maintained at 0.5 mL/min, and the sample volume injected was 2 μL. MS/MS spectra were acquired using information-dependent acquisition mode in Xcalibur software. The electrospray ionization (ESI) source conditions were as follows: sheath gas flow rate at 50 Arb, auxiliary gas flow rate at 15 Arb, capillary temperature at 320 ℃, full MS resolution at 60,000, MS/MS resolution at 15,000, collision energy at SNCE 20/30/40, spray voltage at 3.8 kV (positive) or -3.4 kV (negative). Quality control samples were prepared by mixing all the samples and were inserted into the sample queue to monitor and evaluate the stability of the system and the reliability of the experimental data.

The raw MS data were converted to the mzXML format using ProteoWizard software. Subsequent peak detection, extraction, alignment, and integration were conducted in R using the XCMS package. Metabolite annotation utilized the BiotreeDB MS2 database, and a cut-off annotation of 0.3, as per previous reports 36,37. Statistical analysis was performed in R. Multivariate statistical analyses, including partial least squares discriminant analysis (PLS-DA) and orthogonal projection to latent structures-discriminant analysis (OPLS-DA), were executed using the ropls package. The robustness of the models was assessed through cross-validation and response permutation tests. The variable importance in the projection (VIP) value of each variable was obtained based on the OPLS-DA model. Significantly differentially expressed metabolites were identified when VIP > 1 and P-value < 0.05. For pathway enrichment analysis, the Kyoto Encyclopedia of Genes and Genomes database was used to identify metabolic pathway enrichment. Additionally, metabolic set enrichment analysis (MSEA) was performed using the Small Molecule Pathway Database and analyzed with the MESAp package.

### Transcriptomics study

RNAs were isolated from the livers or RAW 264.6 cells using Trizol Reagent following the manufacturer's instructions. A cDNA library was prepared with the NEBNext Ultra RNA Library Prep Kit for Illumina. The resulting cDNA library was pooled and sequenced on the Novaseq 6000 (Illumina, CA, USA), generating 150-bp paired-end reads. HISAT 2.2.1 was employed to map the sequencing reads to the reference mouse genome GRCm39 38. FeatureCounts 39 was used to obtain read counts per gene locus. Statistical analysis was performed in R, using the Limma package to identify significantly differentially expressed genes (DEGs) with a P-value < 0.05 and a fold change > 1.5. For gene set enrichment analysis (GSEA), the MSigDB Gene annotation gene set was employed via GSEABase. Gene ontology enrichment analysis was conducted using the clusterProfiler package^40^. Additionally, TRRUST transcription factor enrichment analysis was performed using Enricher.

### ATAC-seq and chromatin immunoprecipitation sequencing (ChIP-seq) study

For ATAC-seq, cells (5 x 10^4^) were washed twice in cold PBS and resuspended in 50 μL of cold lysis buffer (10 mM Tris-HCl, pH 7.4, 10 mM NaCl, 3 mM MgCl_2_, 0.1% Tween, 0.1% NP40, and 0.01% Digitonin)^41^. Lysates were centrifuged (750 x g, 10 min, 4 °C), and nuclei were resuspended in 50 μL of transposition reaction mix with 5 μL Tn5 Transposase (TruePrep DNA Library Prep kit V2 for Illumina) and incubated for 30 min at 37 °C. Transposed DNA fragments were purified using a QIAGEN Reaction MiniElute Kit, barcoded with Novo NGS Index Kit for Illumina, and amplified to 240 fmol by NEBNext Ultra II Q5 Master Mix. PCR products were purified using a PCR Purification Kit for paired-end sequencing on Novaseq 6000.

The raw reads generated by ATAC-seq and ChIP-seq were trimmed with Trim-Galore and aligned to the mm10 reference genome using Bowtie2 42. PCR duplicates and multiple mapped reads were removed with Sambamba. Peak calling was performed with MACS2 43, and enriched transcription factor motifs were analyzed through HOMER motif analysis. Statistical analysis was conducted by DESeq2 44. The data were normalized to reads per kilobase per million (RPKM) mapped reads and visualized using Integrative Genomics Viewer (IGV). Signal density surrounding the transcription start site (TSS) was computed and visualized with DeepTools.

### Bioinformatic analysis of scRNA-seq

scRNA-seq data of ATMs was obtained from GEO: GSE168278 18. The Seurat R package was used for analysis. Initial quality control was performed by excluding genes expressed in fewer than three cells and excluding cells based on the following criteria: cells expressing fewer than 200 or more than 2,500 genes, and cells with mitochondrial gene expression greater than 5%.

To analyze macrophage polarization states, we utilized updated gene signatures for M1 and M2 macrophages obtained from the COATES gene set files. The M1 macrophage signature was sourced from COATES_ MACROPHAGE_ M1_ VS_M2_ UP.v2024.1.Mm.gmt and the M2 signature from COATES_ MACROPHAGE_ M1_ VS_M2_DN.v2024.1.Mm.gmt. AddModuleScore function was used to calculate gene enrichment scores for individual cells.

### ChIP

ChIP assays were performed in RAW 264.6 cells with modifications as previously reported 45. Five million cells were fixed in 1% formaldehyde for 8 min at room temperature and then quenched with 1.25 mM glycine for 5 min. The cytoplasm was removed using a swelling buffer (50 mM HEPES-KOH, pH 7.5, 140 mM NaCl, 1 mM EDTA, 10% glycerol, 0.5% NP40, 0.25% Triton X-100, protease inhibitor cocktail) for 10 min on ice, and the nuclei were lysed in lysis buffer (50 mM Tris-HCl, pH 8.0, 10 mM EDTA, 1% SDS, protease inhibitor cocktail) for 10 min on ice. DNA was fragmented to an average size of 500 bp using a Bioruptor sonicator (Diagenode, Seraing, Belgium). The lysate was then diluted 10-fold with dilution buffer (10 mM Tris-HCl, pH 8.0, 100 mM NaCl, 1 mM EDTA, 0.5 mM EGTA, 1% Triton X-100, 0.1% deoxycholate, protease inhibitor cocktail), and 3 µg of anti-C/EBPβ or mouse IgG antibody was added and incubated overnight. Protein G Dynal beads were used to collect the complex, which was subsequently washed three times with wash buffer (50 mM HEPES-KOH, pH 7.0, 0.5 M LiCl, 1 mM EDTA, 0.7% deoxycholate, 1% NP40). Finally, the DNA was eluted, de-crosslinked, and purified by QIAquick PCR Purification Kit. Real-time PCR was performed using qPCR SYBR Green Master Mix with the following PCR program: 95 °C for 1 min, followed by 40 cycles of 95 °C for 15 s, 60 °C for 20 s, and 72 °C for 40 s. The primer sequences are provided in **Table S1**.

### Induced fit docking

The two sections of C/EBPβ, 1h8a and 1h89, were subjected to docking using the existing structure of esculetin. Both methods adhered to the same protocol, utilizing AutoDock's induced fit docking pipeline 46. The molecular minimization structure was prepared in Chem 3D, and PyMOL was employed for model pretreatment and removal of water molecules. AutoDock facilitated protein preparation for docking by adding hydrogen, eliminating water molecules within the active site, and generating a protonated state at pH 7 (±1). Alongside adjusting protonation states, AutoDock optimized hydrogen bonds. The Grid Box module enveloped the entire protein, with an active pocket that interfaces for induced fit. The docking site of the target was derived from the ligand binding site in the eutectic structure. The number of docking conformations of ligands was set to 20, and the truncation distance of clustering was set to 0.5 Å. Top docking poses were selected based on the docking score of AutoDock Vina and the visual inspection of PyMOL to confirm the probable binding mode.

### Quantitative Real-Time Reverse Transcription PCR (qRT-PCR)

qRT-PCR was conducted using the RNA-direct SYBR Green Real-Time PCR Master Mix. Glyceraldehyde 3-phosphate dehydrogenase (GAPDH) was utilized as the internal control. The primer sequences are provided in Table S1.

### Cytokine/ chemokine array

Two hundred fifty microliters of serum from mice, diluted to 500 μL with ddH₂O, were analyzed using a Cytokine Array C1 kit (AAM-CYT-1-8) according to the manufacturer's instructions. The chemiluminescent signal was visualized with a ChemiDoc MP imaging system (Bio-Rad).

### Histological analysis

The eWAT and livers were fixed in 4% paraformaldehyde and subsequently embedded in paraffin. Paraffin-embedded tissues were subjected to deparaffinization and rehydration. For immunohistochemical (IHC) staining, sections were treated with 3% H_2_O_2_ for 25 min to block endogenous peroxidase activity. Non-specific binding sites were blocked by incubating sections with 3% BSA in PBS for 30 min. Subsequently, sections were incubated with primary antibodies against F4/80 and CD36 (1:500 dilution) overnight at 4 °C. Detection was performed using peroxidase-conjugated secondary antibodies and DAB. After the secondary antibody incubation, slides were counterstained with hematoxylin for 3 min, followed by dehydration and mounting. For hematoxylin and eosin (H&E) staining, paraffin-embedded tissues of livers and eWAT were stained with a hematoxylin and eosin kit according to the manufacturer's instructions. Stained sections were examined, and images were captured using a bright-field microscope (TissueFAXS P1, Tissuegnostics, Vienna, Austria).

### Western blot

Equal amounts of protein were loaded onto sodium dodecyl sulfate-polyacrylamide gel electrophoresis (SDS-PAGE) and subsequently transferred onto polyvinylidene fluoride (PVDF) membranes (Bio-Rad, catalog #1620177) following standard procedures. The membranes were blocked in Tris-buffered saline with 0.5% Tween 20 and 3% skim milk at room temperature for 2-3 h. This was followed by overnight incubation with primary antibodies at 4 °C. After thorough washing, membranes were then incubated with horseradish peroxidase (HRP)-conjugated secondary antibodies at room temperature for 2-3 h. Protein bands were visualized using Western Bright ECL HRP substrate in a ChemiDoc MP imaging system (Bio-Rad). Details regarding the antibodies used can be found in Table S1. Whole gel images shown in Figure S7.

### Terahertz chemical microscope assay

A C/EBPβ-immobilized sensing plate was prepared as follows, based on our previous reports 47,48. Briefly, a silicon-on-sapphire plate containing 500 μm Al_2_O_3_, 500 nm Si, and a SiO_2_ surface layer, was ultrasonically cleaned with 99.5% acetone and 99.5% ethanol (2 min each) to remove surface contaminants and sterilize. The SiO_2_ surface was introduced to hydroxyl and amino groups by incubating with 30 μL of 200 mM NaOH (5 minu) followed by 30 μL of 2% (3-aminopropyl) triethoxysilane (APTES) (30 min). Next, 120 μL of 1 μg/mL recombinant C/EBPβ protein was added to the wells and incubated at 25 °C for 6 h and kept overnight for reaction at room temperature to generate the C/EBPβ-immobilized sensing surface. To prevent nonspecific binding, 0.1 M ethanolamine-HCl was added to all wells for 20 min at room temperature prior to analysis. The C/EBPβ-immobilized plate was then loaded into a terahertz chemical microscope (Homemade, Okayama university) and terahertz amplitude measured following addition of varying concentrations of esculetin.

### Statistical analysis

Statistical analysis was performed with Prism 9 software. Data are shown as meanSD. Statistical tests performed for each dataset are described within the relevant figure legends. P-values ≤0.05 were considered statistically significant. P-values are denoted in figures as follows: ns, not significant; p > 0.05; *, p < 0.05; **, p < 0.01; and ***, p < 0.001.

## Results

### Esculetin elevates the levels of postprandial serum HDL cholesterol

Mice were administered lipid-rich meals for only 3 days to investigate the effects of esculetin or statins on postprandial lipid levels (Figure 1A). First, we validated that esculetin treatment at doses of 90 and 180 mg/kg/d did not significantly alter serum levels of the liver enzymes alanine aminotransferase and aspartate transaminase (Figure S1B) or cause histological changes in liver tissue of high fat-fed mice (Figure S1C). Further, both 90 and 180 mg/kg esculetin did not significantly alter expression of hepatic apoptosis-associated genes (Figure S1D) and transcriptomic profiling showed apoptosis pathways were not enriched among esculetin-induced changes (Figure S1E). Together with previous reports 49, these data suggest esculetin does not exhibit liver toxicity at the tested doses.

The non-fasting blood serum was analyzed to assess the lipid profile after a high-fat meal. Esculetin treatment resulted in a significant increase in postprandial HDL cholesterol levels, from 2.554 ± 0.744 mmol/L to 3.963 ± 0.425 or 3.707 ± 0.682 mmol/L at doses of 90 or 180 mg/kg/d, respectively. In contrast, pitavastatin treatment at a high dose of 1.55 mg/kg/d yielded a relatively modest increase in HDL cholesterol levels to 3.360 ± 0.146 mmol/L (Figure 1B). Esculetin at a dose of 30 mg/kg/d or simvastatin at a dose of 7.6 mg/kg/d did not result in significant alterations in postprandial HDL levels (Figure 1B). Simvastatin, but not esculetin or pitavastatin, significantly decreased postprandial LDL levels (Figure 1C). Treatment with esculetin or stains did not significantly alter postprandial cholesterol and triglyceride in HFD-fed mice (Figure 1D-E). These findings suggest that esculetin can increase postprandial HDL cholesterol levels, but not alter the LDL cholesterol levels.

### Esculetin promotes cholesterol excretion

UHPLC-MS/MS was utilized for the identification of serum metabolites to investigate the impact of esculetin on postprandial metabolic profiles in serum. *Pearson* correlation analysis of quality control samples confirmed the relative stability of the UHPLC-MS/MS system (Figure S2A). PLS-DA and OPLS-DA plots, generated for both positive and negative ion models, indicated that esculetin induced alterations in postprandial metabolic profiles (Figure S2 B-C). The regression lines of Q^2^ and R^2^Y, based on cross-validation and permutation testing of the PLS-DA and OPLS-DA models for both ion models, affirmed the reliability and accuracy of these models in capturing significantly differentially expressed metabolites (DEMs) influenced by esculetin (Figure S2D-E).

Esculetin elicited significant changes in 110 serum metabolites in HFD-fed mice (Figure S2F), including 33 lipid and cholesterol-associated metabolites such as the metabolic products of phosphatidylcholine, phosphatidylethanolamine and triglycerides (Figure S2G). Metabolite set enrichment and KEGG analysis revealed that esculetin-induced DEMs were enriched in cholesterol excretion, including bile acid biosynthesis and ABC transporters 50, and lipid metabolism such as mitochondrial beta-oxidation of long-chain saturated fatty acids, fatty acid elongation in mitochondria, fatty acid metabolism, steroid biosynthesis, fatty acid biosynthesis, glycerolipid metabolism, biosynthesis of unsaturated fatty acids, and fatty acid degradation (Figure 1F-G). Furthermore, we quantified the levels of bile acid in bile, where cholesterol is converted to bile acid for excretion 51. Esculetin significantly increased bile acid content, raising levels from 19.27±8.71 mmol/L to 35.88±8.08 mmol/L and 51.77±3.31 mmol/L at doses of 90 and 180 mg/kg/d in HFD-fed mice, respectively. Pitavastatin at the dose of 1.55 mg/kg/d elevated bile acid levels to 46.06±6.61 mmol/L, while simvastatin had no effect. Additionally, the cytochrome P450 family gene *Cyp7b1* was significantly upregulated by esculetin without altering the expression of *Cyp7a1* (Figure 1I) , both of which encode rate-limiting enzymes for bile acid synthesis 52. In contrast, the expression levels of *Apoa1*, a major apolipoprotein component of HDL particles, were not significantly altered (Figure S2H). These results suggest that esculetin not only elevates postprandial HDL cholesterol levels but also promotes cholesterol excretion.

### Esculetin elevates HDL levels in an eWAT-dependent manner by promoting CD36-mediated phagocytosis in ATMs

**Figure 2:**
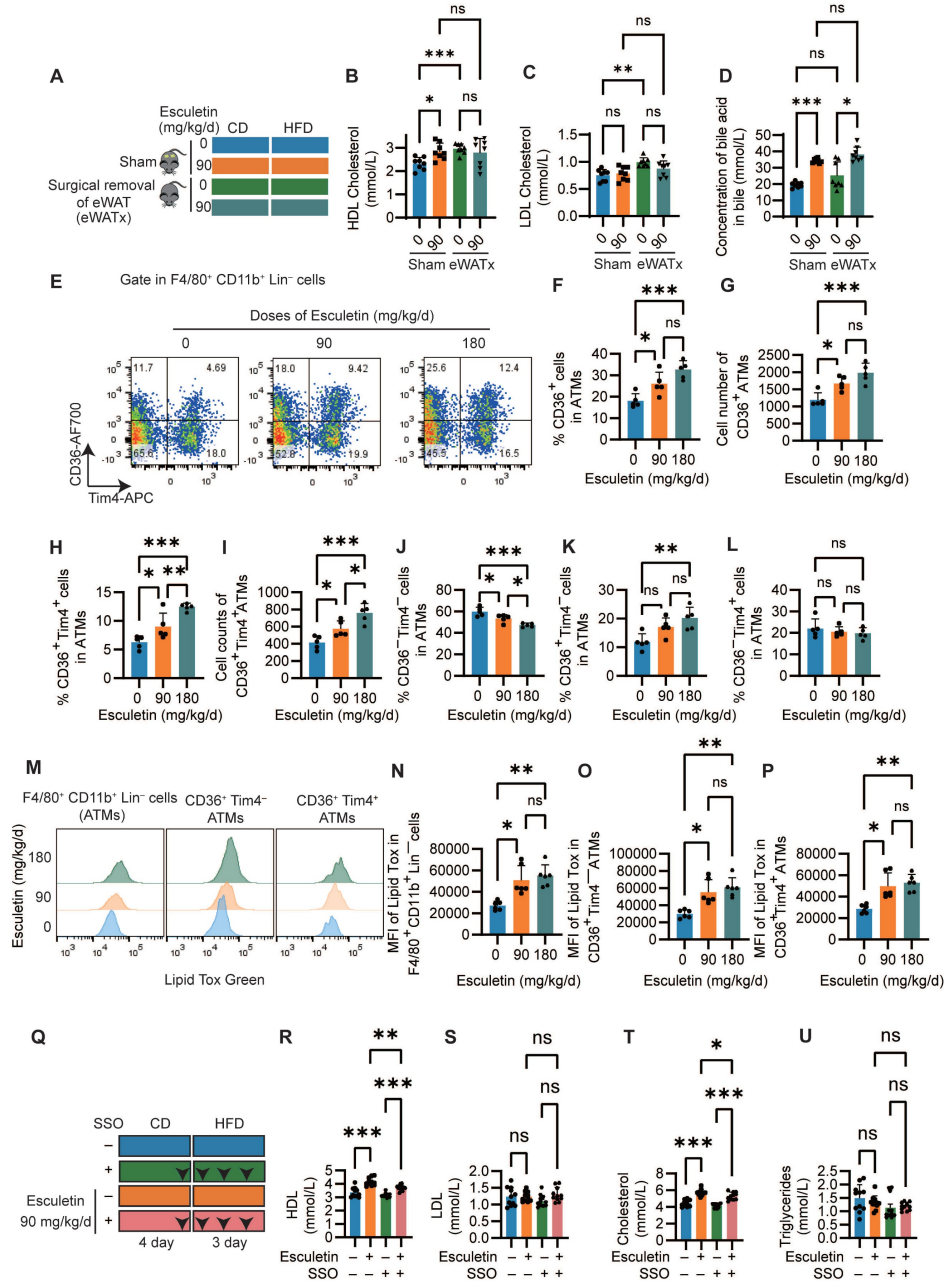
** Esculetin elevates HDL levels in an eWAT-dependent manner by promoting CD36-mediated phagocytosis in ATMs. (A)** Schematic representation of the experimental design. Sham surgery and epididymal white adipose tissue removal (eWATx) were performed on the mice. The mice were then treated with either a 0 or 90 mg/kg/day of esculetin for 7 days, along with 3 days of high-fat diet feeding as above. **(B, C)** Quantification of postprandial serum lipids. **(D)** Bile acid levels in bile were quantified. Data points represent individual mice (n = 8) across two independent experiments. The P values were calculated using the Brown-Forsythe ANOVA test with Dunnett's T3 multiple comparisons test (B, D), one-way ANOVA with Tukey's multiple comparisons test (C). (**E**) Representative plots indicating CD36 and Tim4-defined ATM subsets. (**F-L**) Quantification of frequency and cell counts per mouse of the indicated ATM subsets. Data points in graphs represent individual mice (n = 5) over two independent experiments, analyzed by one-way ANOVA with Tukey's multiple comparisons test. (**M**) Representative flow cytometry plots show the intensity of LipidTOX in ATM subsets. (**N-P**) Quantification of LipidTOX intensity in specified ATM subsets. Data points in graphs represent individual mice (n = 6) over two independent experiments, analyzed by Kruskal-Wallis test with Dunn's multiple comparisons. (**Q**) Schematic of SSO and esculetin administration timeline. **(R-U)** Serum HDL, total cholesterol, LDL cholesterol and triglycerides. Data points in graphs represent individual mice (n = 11) over two independent experiments analyzed by one-way ANOVA with Tukey's multiple comparisons test.

Since visceral adipose tissue, specifically eWAT, have been reported to be involved in postprandial HDL cholesterol metabolism 18,33, we first confirmed the role of eWAT in esculetin-induced increases in postprandial HDL by surgically removing eWAT (Figure 2A). Meanwhile, adipose tissue serves as a highly active and efficient metabolic buffer, playing a crucial role in regulating daily dietary lipid flux in circulation 10. The removal of adipose tissue typically leads to fluctuated serum lipid including HDL levels due to the loss of this critical buffering capacity. Therefore, the comparison within the eWAT-removal group to assess esculetin's effects remains more meaningful and relevant. In the sham group, esculetin significantly elevated serum HDL levels, whereas this increase was not observed in mice without eWAT (Figure 2B). LDL, total cholesterol, and triglyceride levels did not significantly change in any of the groups (Figure 2C, Figure S3A-B). The bile acid concentration in bile significantly increased after esculetin administration in both the sham and eWAT-removal groups (Figure 2D). The results suggest that esculetin elevates postprandial HDL cholesterol in an eWAT-dependent manner and promotes cholesterol excretion independently of eWAT.

H&E staining of eWAT showed that adipocyte size did not significantly change after HFD feeding or esculetin treatment (Figure S3C-D). The number and distribution of F4/80^+^ macrophages also did not differ significantly among groups (Figure S3C, E). However, CD36^+^ cells were observed in adipose tissue, and esculetin administration significantly increased the number of CD36^+^ cells in HFD-fed mice (Figure S3C, F). Thus, esculetin elevates postprandial HDL cholesterol in an eWAT-dependent manner, which is associated with an increase in CD36^+^ cells in eWAT.

Given the observed alterations in ATM subsets following esculetin administration, systemic inflammation and the some adipokines was analyzed. A total of 22 cytokines or chemokines were semi-quantified using a membrane array with serum from HFD-fed mice treated with either 0 or 90 mg/kg/d of esculetin. There were no apparent alterations in the expression of the tested cytokines and chemokines in HFD-fed mice after esculetin administration, indicating that esculetin plays a minor role in these short-term HFD-fed mice (Figure S4A). Flow cytometry was next used to analyze effects of esculetin on ATM subsets in eWAT. Total ATMs defined as CD45^+^ F4/80^+^CD11b^+^ Lineage (CD19, TCRb, Ly-6g, Ly-6c) cells were not significantly altered following esculetin administration (Figure S3G-I, Figure S4B). However, treatment with 90 or 180 mg/kg/d esculetin significantly increased both the percentage and absolute counts of CD36^+^ ATMs (Figure 2G-I). Moreover, the ratios and counts of CD36^+^Tim4^+^ ATMs were also significantly elevated by esculetin (Figure 2H-L), while the ratios of CD36 Tim4 ATMs were decreased at both doses (Figure 2J). The percentage of CD36^+^ Tim4 cells was significantly increased by 180 mg/kg esculetin (Figure 2K). The CD36 Tim4^+^ subset remained unchanged across groups (Figure 2L). Collectively, these data indicate esculetin promotes enrichment of a CD36^+^ ATM subset previously shown to regulate postprandial HDL metabolism and cholesterol uptake 18,23.

**Figure 3:**
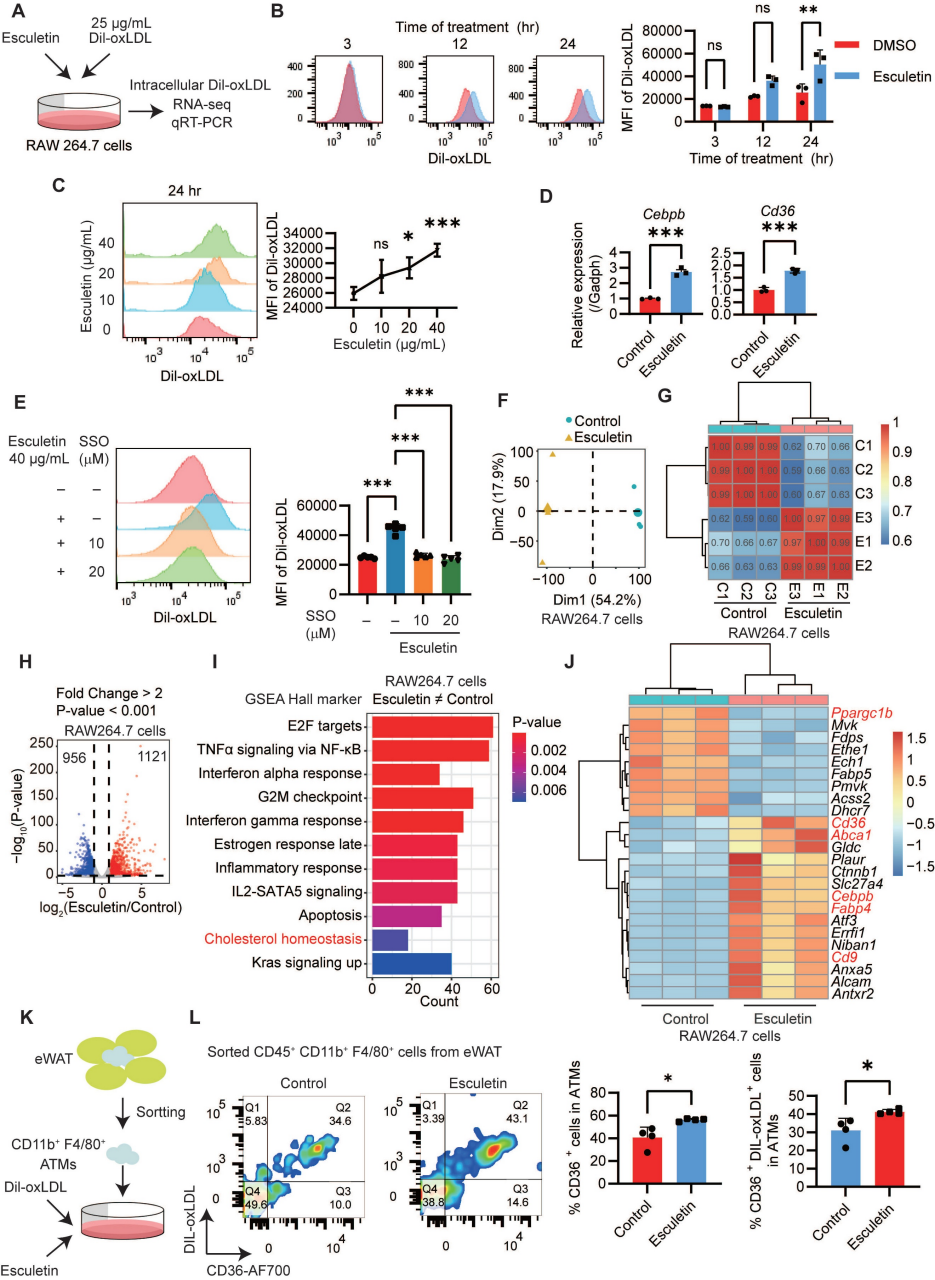
** Esculetin enhances CD36-mediated phagocytosis of macrophages *in vitro*. (A)** Schematic depiction of the experimental setup. RAW264.7 cells were treated with 25 g/mL DIL-oxLDL and esculetin at the indicated concentrations for 3 to 24 h. (**B and C**) Quantification of DIL-oxLDL uptake by flow cytometry at specified timepoints and doses in RAW264. 7 cells. Data points in graphs represent individual independent experiments (n = 3) analyzed by two-way ANOVA with Šídák's multiple comparisons test (B) or one-way ANOVA with Dunnett's multiple comparisons test (C). (**D**) qRT-PCR analysis of *Cd36* and *Cebpb* mRNA expression. Data points in graphs represent individual independent experiments (n = 3) analyzed by unpaired t test. (**E**) DIL-oxLDL uptake following SSO treatment. Data points in graphs represent individual independent experiments (n = 5) analyzed by one-way ANOVA with Tukey's multiple comparisons test. (**F-J**) RNA sequencing of RAW264.7 cells treated with DIL-oxLDL as well as 0 or 40 μg/mL esculetin for 24 h (n = 3). (**F**) Principal component analysis (PCA) plot. (**G**) *Pearson* correlation coefficients among all the RAW264.7 cells treated with or without esculetin. (**H**) Volcano plot of esculetin-induced significantly differentially expressed genes (DEGs). (**I**) Gene set enrichment analysis (GSEA). (**J**) Heatmap of esculetin-induced cholesterol homeostasis-associated DEGs. **(K)** CD11b^+^ F4/80^+^ cells were sorted from eWAT and treated with 25 g/mL DIL-oxLDL and either 0 or 40 µg/mL esculetin for 24 h. **(L)** The percentage of the indicated cell population was quantified by flow cytometry, and representative flow cytometry plots are shown. Data points represent individual experiments (n = 4), analyzed using an unpaired t-test.

Moreover, intracellular lipid content scavenged by ATMs was also examined using LipidTox staining. Esculetin treatment at doses of 90 and 180 mg/kg/d significantly increased the fluorescence intensity of LipidTox in total macrophages as well as in the CD36^+^ Tim4^+^ and CD36^+^ Tim4 macrophage subsets (Figure 2M-P). In contrast, neither the percentage of CD36^+^ macrophages nor LipidTox intensity was altered in hepatic macrophages (Figure S4C-D). Furthermore, the CD36 inhibitor SSO was administered during esculetin treatment in high fat-fed mice (Figure 2Q). This abolished esculetin-induced increases in postprandial HDL and total cholesterol, while LDL and triglycerides were unaffected (Figure 3R-U). Together, these findings indicate esculetin enhances the phagocytic capacity of CD36^+^ ATMs to promote postprandial HDL biogenesis.

### Esculetin enhances CD36-mediated phagocytosis of macrophages in vitro

To understand the mechanisms underlying the enhancement of macrophage phagocytosis by esculetin, DIL-ox-LDL-treated RAW264.7 macrophages were used in an *in vitro* experiment (Figure 3A). Firstly, it was observed that esculetin significantly increased the fluorescence intensity of intracellular DIL-ox-LDL in a dose- and time-dependent manner (Figure 3B-C). Additionally, the expression levels of *Cd36* and *Cebpb,* which are associated with phagocytosis of macrophages 53,54*,* were significantly increased after the addition of esculetin in ox-LDL-treated RAW264.7 cells (Figure 3D). SSO could significantly repress esculetin-induced increased uptake of ox-LDL *in vit*ro (Figure 3E). These findings suggest that esculetin enhances the phagocytosis of RAW264.7 cells via CD36 *in vitro*.

The transcriptomic alterations induced by esculetin were further analyzed in ox-LDL-treated RAW264.7 cells. Principal component analysis (Figure 3F) and *Pearson* correlation analysis (Figure 3G) indicated that esculetin induces transcriptomic alterations in ox-LDL-treated RAW264.7 cells. A total of 1121 genes were significantly upregulated, while 956 genes were significantly downregulated after the addition of esculetin in RAW264.7 cells (Figure 3H). The DEGs induced by esculetin were enriched in pathways related to cholesterol homeostasis including* Cd36, Abca1, Slc27a4, Cebpb, Fabp4* and so on (Figure 3-J). Furthermore, ATMs were sorted and treated with esculetin and DIL-ox-LDL *in vitro* (Figure 3K, Figure S4E). Compared to the control group, the addition of esculetin significantly increased the percentage of CD36^+^ cells and CD36^+^ DIL-ox-LDL^+^ cells in sorted ATMs (Figure 3L). The results suggested that esculetin enhances CD36-meidated phagocytosis of macrophages and regulate cholesterol homeostasis* in vitro*.

### Esculetin regulates the CD36-meidated phagocytosis of macrophages involved in C/EBPβ

**Figure 4:**
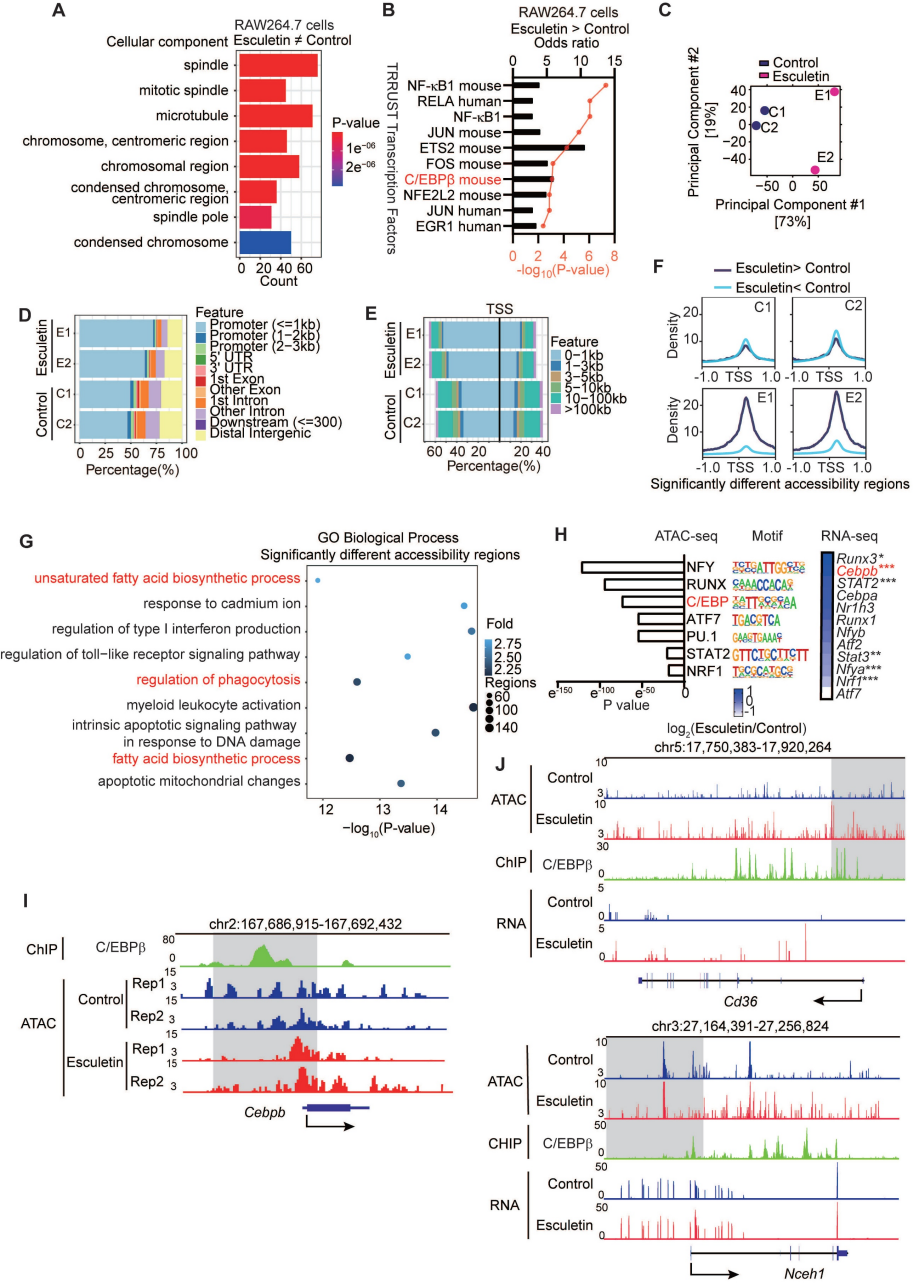
** Esculetin Modulates C/EBPβ-Mediated Epigenetic Landscape Alterations. (A)** Gene ontology (GO) term enrichment analysis of esculetin-induced significantly differentially expressed genes (DEGs) in DIL-oxLDL-treated RAW264.7 cells was performed.** (B)** Esculetin-induced significantly upregulated genes in DIL-oxLDL-treated RAW264.7 were used for enrichment analysis against TRRUST transcription factor gene sets. **(C-J)** ATAC-seq was conducted using RAW264.7 cells stimulated with 25 µg/mL DIL-oxLDL and either 0 or 40 µg/mL esculetin for 24 h (n = 2). **(C)** Principal component analysis (PCA) plot. **(D and E)** The peak distribution among typical regions in individual samples. **(F)** The peak distribution of esculetin-induced significantly different accessibility regions. **(G)** GO term enrichment analysis of esculetin-induced significantly different accessibility regions. **(H)** Homer motif enrichment of esculetin-induced significantly different accessibility regions and the heat map of the enrichment motif-associated genes. **(I)** The representative plot of ATAC-seq and Chip-seq of C/EBP on the loci of *Cebpb*.** (J)** The representative plot of ATAC-seq, RNA-seq, and ChIP-seq of C/EBP for *Cd36* and *Nceh1*. The Chip-seq of C/EBP data was obtained from GSE173970.

To understand the potential targets of esculetin in regulating macrophage phagocytosis and cholesterol homeostasis, we performed GO term enrichment analysis of esculetin-induced DEGs in RAW264.7 cells. The results indicated that esculetin could modulate pathways associated with chromosome-related processes and condensed chromosomes, suggesting the involvement of epigenetic mechanisms in esculetin's regulation of CD36-mediated macrophage phagocytosis (Figure 4A). Notably, a key transcription factor, C/EBPβ, controlled genes that were enriched in esculetin-induced upregulated genes (Figure 4B).

Subsequently, ATAC-seq was employed to unravel esculetin-induced alterations in the epigenetic landscape during macrophage phagocytosis in ox-LDL-treated RAW264.7 cells. PCA suggested that esculetin induced an alteration in the epigenetic landscape (Figure 4C), with open chromatin regions primarily located in promoter regions (Figure 4D) and around transcription start sites (TSS) (Figure 4E). The ratios of open chromatin regions around promoters and TSS tended to increase after esculetin treatment, and esculetin-induced significantly upregulated peaks were concentrated near TSS regions (Figure 4D-F). Moreover, we observed that esculetin-induced significantly different accessibility regions were enriched in pathways related to the regulation of phagocytosis and lipid metabolism (Figure 4G). Therefore, these results suggest that esculetin induces alterations in the epigenetic landscape, which may control macrophage phagocytosis and lipid metabolism.

Motif enrichment analysis revealed significant overrepresentation of binding sites for NFY, RUNX, and C/EBP family proteins among esculetin-altered accessible chromatin regions (Figure 4H). Notably, the gene *Cebpb*, encoding the C/EBPβ transcription factor involved in lipid metabolism and macrophage phagocytosis regulation, was upregulated in oxLDL-treated RAW264.7 cells (Figure 4H). Open chromatin regions near the *Cebpb* TSS were increased by esculetin treatment, and C/EBPβ binds to this region (Figure 4I). C/EBPβ also can bind upstream of *Cd36* and *Nceh1*, two genes exhibiting both esculetin-induced chromatin accessibility changes and transcriptional upregulation in oxLDL-exposed RAW264.7 cells (Figure 4J). These findings collectively suggest that esculetin regulates the CD36-mediated phagocytosis of macrophages, and this process involves C/EBPβ and epigenetic landscape alterations.

### Esculetin enhances CD36-mediated phagocytosis of macrophages via C/EBPβ

**Figure 5:**
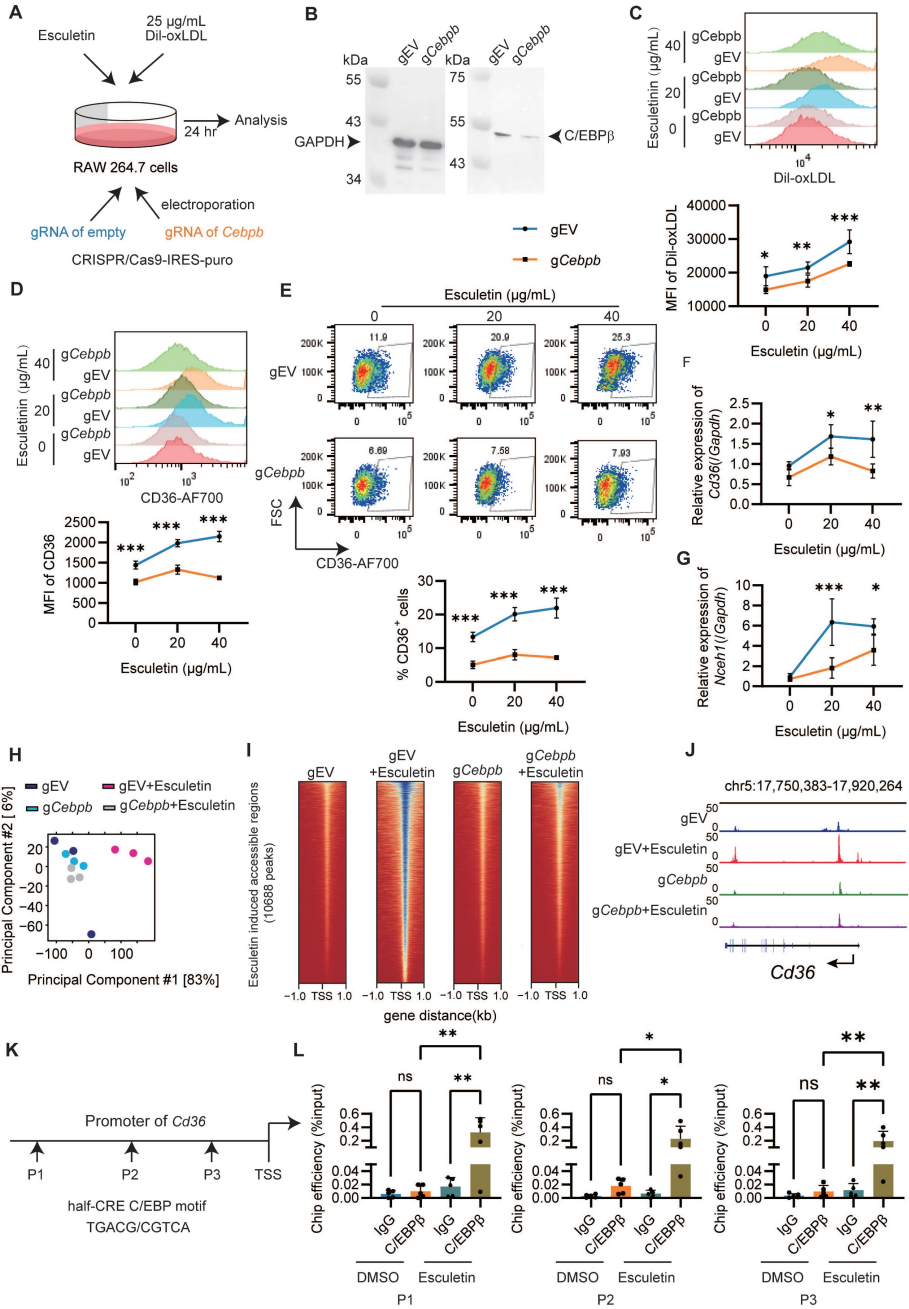
** Esculetin enhances the CD36-mediated phagocytosis of macrophages via C/EBPβ. (A)** Schematic depiction of the experimental setup.** (B)** Western blot of C/EBPβ knockdown efficiency in RAW264.7 cells transfected with empty or *Cebpb* gRNA. One representative out of three similar experiments is displayed.** (C)** Representative flow cytometry plots and quantification of DIL-oxLDL intensity in RAW264.7 cells. (**D**) Representative flow cytometry plots and quantification of CD36 intensity in RAW264.7 cells. (**E**) Percentage of CD36^+^ RAW264.7 cells. mRNA expression of *Cd36* (**F**) and *Nceh1* (**G**) by qRT-PCR. Graphs show mean ± SD of n = 3 experiments, analyzed by two-way ANOVA with Šídák's test.** (H-J)** ATAC-seq was conducted using RAW264.7 cells transfected with empty or *Cebpb* gRNA stimulated with 25 µg/mL DIL-oxLDL and either 0 or 40 µg/mL esculetin for 24 h (n = 3)**. (H)** PCA plot. **(I)** The peak distribution heatmap of esculetin-induced accessible regions.** (J)** The representative plot of ATAC-seq on the loci of *Cd36.* **(K)** The primer sets targeted loci on the promoter region of *Cd36* were used for the C/EBPβ ChIP assay. **(L)** The binding efficiency of C/EBPβ at the indicated loci of *Cd36* was quantified by qPCR. Data points represent individual experiments (n = 5), analyzed using one-way ANOVA with Holm-Šídák's multiple comparisons test.

To confirm the essential role of C/EBPβ in esculetin's regulation of CD36-mediated macrophage phagocytosis, RAW264.7 cells deficient in *Cebpb* were created using CRISPR/Cas9 (Figure 5A-B). In *Cebpb*-deficient cells, esculetin-induced increased uptake of ox-LDL (Figure 5C), the expression of CD36 (Figure 5D), the percentage of CD36^+^ cells (Figure 5E), and the transcription of *Cd36* (Figure 5F) and *Nceh1* (Figure 5G) were significantly repressed in ox-LDL-treated RAW264.7 cells.

Subsequently, we performed ATAC-seq analysis in RAW264.7 cells transfected with empty or *Cebpb* gRNA to examine C/EBPβ-mediated changes in the epigenetic landscape induced by esculetin. PCA revealed that C/EBPβ knockout reversed the esculetin-induced alterations in the epigenetic landscape (Figure 5H). The heatmap of peaks distribution demonstrated that C/EBPβ knockout significantly reduced the number of chromatin-accessible regions increased by esculetin treatment (Figure 5I). Notably, the esculetin-induced increase in chromatin accessibility at the *CD36* gene locus was markedly diminished after C/EBPβ knockout (Figure 5J). Together, these results indicate that C/EBPβ ablation reverses the epigenetic modifications induced by esculetin, highlighting the essential role of C/EBPβ in mediating esculetin's effects on chromatin remodeling and the regulation of lipid metabolism.

Furthermore, the binding of C/EBPβ to the promoter regions of *Cd36* was investigated using chromatin immunoprecipitation in half-CRE (C/EBPβ motif) enriched regions (Figure 5K). After the addition of esculetin, the binding of C/EBPβ to the *Cd36* promoter significantly increased in ox-LDL-treated RAW264.7 cells (Figure 5L), indicating that esculetin could enhance the binding activity of C/EBPβ to *Cd36* promoter. Thus, these findings indicate that esculetin enhances macrophage phagocytosis via C/EBPβ.

### Esculetin could directly bind to C/EBPβ

**Figure 6:**
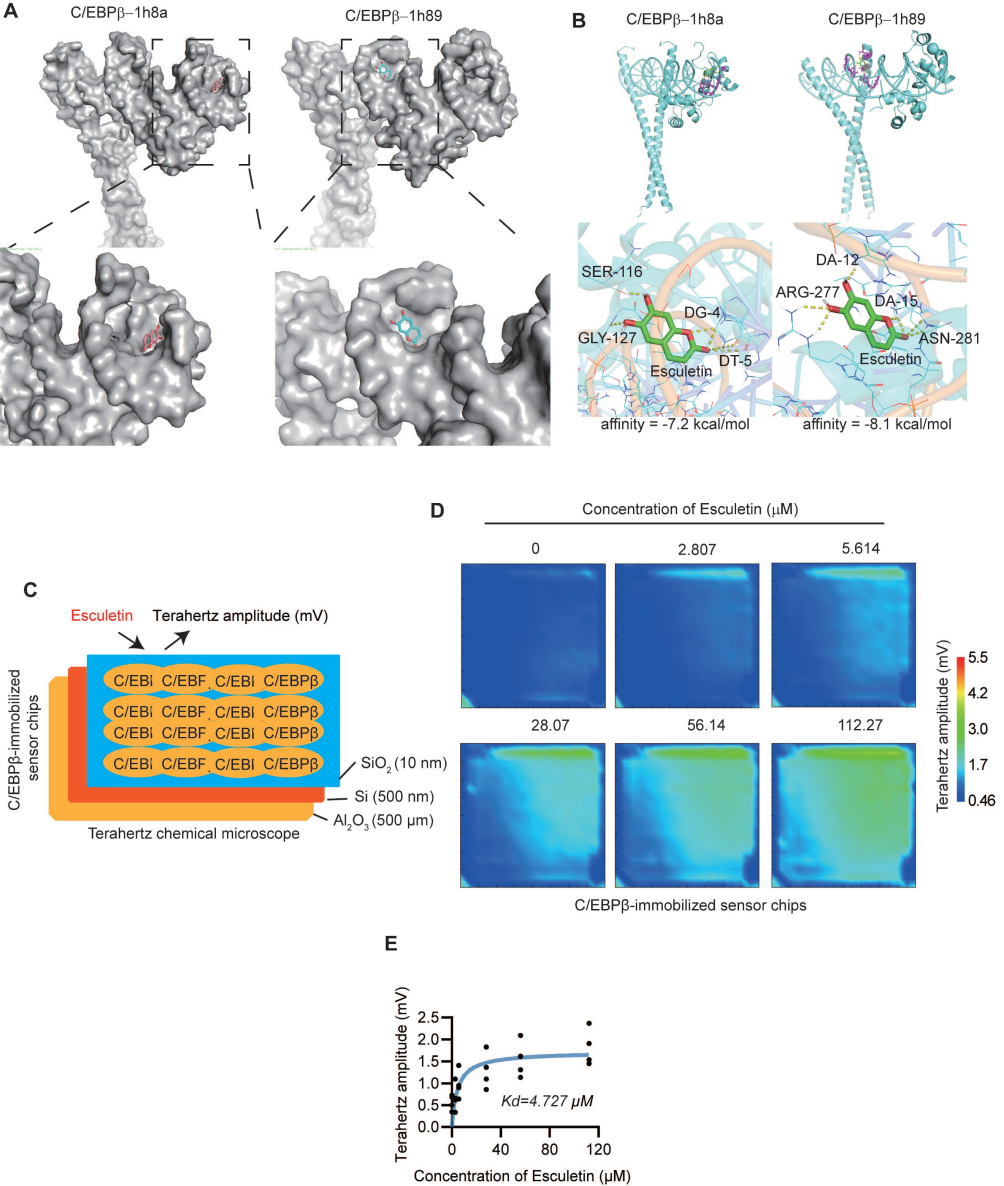
** Esculetin directly interacts with C/EBPβ. (A and B)** Predicted binding modes of esculetin with C/EBPβ (PDB: 1h8a, 1h89). **(C)** Schematic overview of the terahertz chemical microscope assay using a C/EBPβ-immobilized sensor surface.** (D)** Terahertz signals measured on C/EBPβ-immobilized sensor chips with or without the indicated concentrations of esculetin. **(E)** Binding curve analysis of esculetin-C/EBPβ interactions by terahertz chemical microscopy. Kd = 4.727 μM. Data points in graphs represent individual independent experiments (n = 4).

Molecular docking analysis was performed to understand potential binding modes and interactions between esculetin and C/EBPβ (PDB: 1h8a, 1h89). Results showed that esculetin could enter the active site of C/EBPβ and interact with both C/EBPβ and its target DNA, with predicted binding affinities of -7.2 kcal/mol (1h8a) and -8.1 kcal/mol (1h89) (Figure 6A-B). A terahertz chemical microscope assay was then used to monitor esculetin-C/EBPβ binding kinetics in real-time by measuring changes in terahertz amplitude using a C/EBPβ-immobilized sensor 47 (Figure 6B). Addition of increasing concentrations of esculetin resulted in corresponding changes in terahertz amplitude (Figure 6C), indicating concentration-dependent binding to surface-immobilized C/EBPβ. Analysis of binding curves revealed esculetin binds C/EBPβ with relatively high affinity (*Kd* = 4.727 M) (Figure 6D). Together, these results demonstrate esculetin directly interacts with its target, C/EBPβ.

### Esculetin activates C/EBPβ in vivo

**Figure 7:**
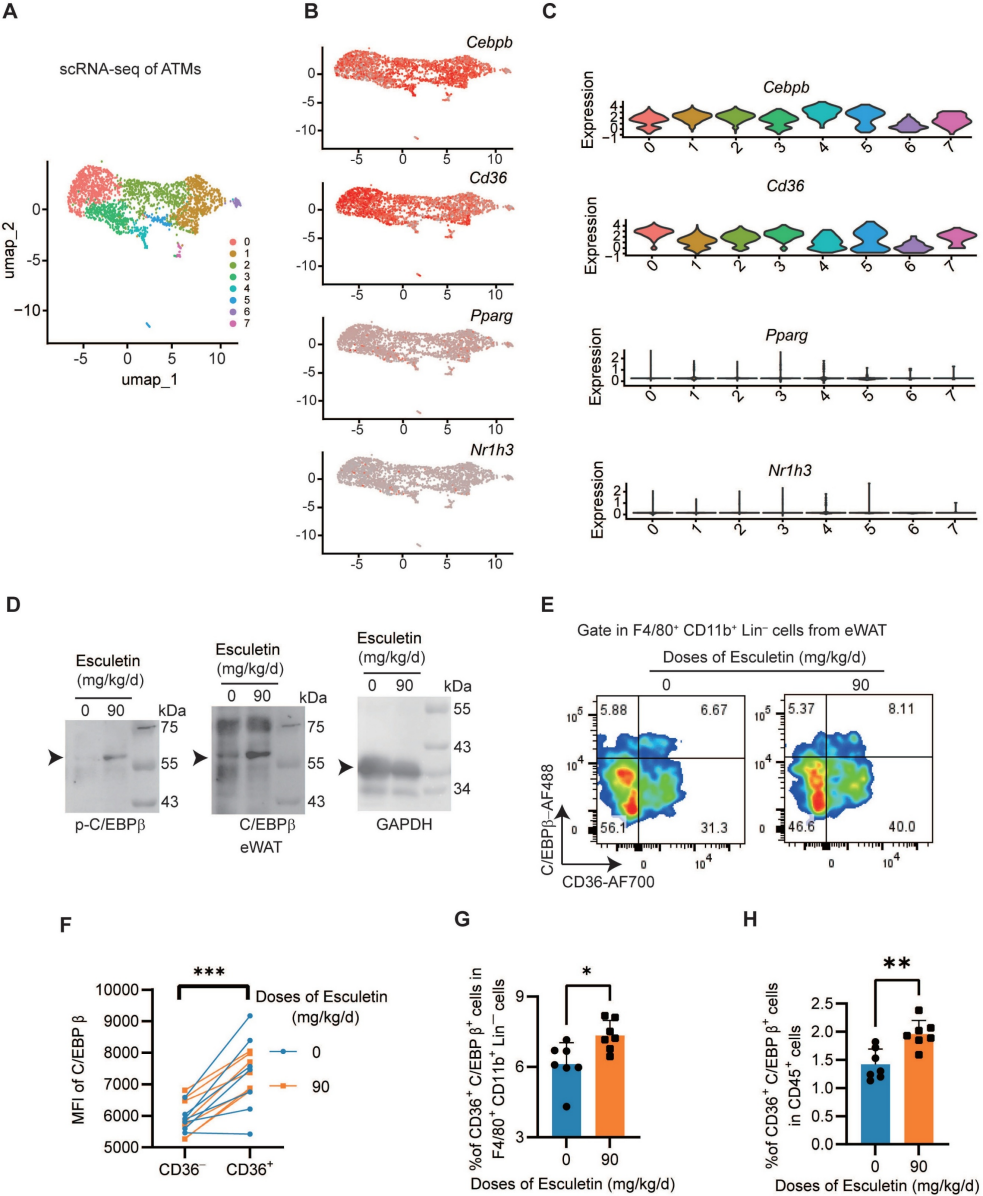
** Esculetin activates C/EBPβ in ATMs. (A)** Unsupervised clustering of ATMs using UMAP, where each dot represents a single cell, colored by cluster assignment. **(B)** Expression profiles of *Cebpb, Cd36, Pparg and Nr1h3* in ATMs. **(C)** Violin plots showing *Cebpb, Cd36, Pparg and Nr1h3* expression by cluster. (**D**) Western blot of C/EBPβ and p-C/EBPβ expression in eWAT of high fat-fed mice with or without esculetin. One representative out of three similar experiments is shown. **(E)** Representative plots indicating CD36 and Tim4-defined ATM subsets, **(F)** The quantification of C/EBPβ intensity in CD36^-^ and CD36^+^ ATMs. Data points in graphs represent individual mice (n = 14) over two independent experiments analyzed by paired t test.** (G, H)** Percentage of CD36^+^C/EBPβ^+^ ATMs in total ATMs and CD45^+^ cells from high fat-fed mouse eWAT with or without esculetin. Data points in graphs show individual mice (n = 7) over 2 experiments, analyzed by unpaired t-test.

To understand the expression profile of *Cebpb* in ATMs, we performed scRNA-seq analysis and identified seven distinct clusters (Figure 7A). *Cebpb* and *Cd36* was universally expressed across the ATMs (Figure 7B-C). In contrast, neither liver X receptor α (LXRα, encoded by *Nr1h3*), a key transcriptional regulator of lipid metabolism 55, nor peroxisome proliferator-activated receptor gamma (PPARγ, encoded by *Pparg*), a critical transcriptional factor promoting CD36 expression in lipid metabolism 56, was expressed in ATMs (Figure 7B-C). The postprandial high-fat diet did not alter the expression patterns of M1/M2 macrophage signature gene sets or characteristic marker genes (Figure S6A-F). Furthermore, in the eWAT of HFD-fed mice, esculetin was found to elevate the expressions of C/EBPβ and the phosphorylation of C/EBPβ (Figure 7D). In CD36^+^ ATMs, the expression of C/EBPβ was significantly higher compared to CD36^-^ ATMs (Figure 7E-F), and esculetin significantly elevated the ratios of CD36^+^ C/EBPβ^high^ ATMs in total ATMs or total CD45^+^ leukocytes (Figure 7G-H). These results suggest that esculetin activates C/EBPβ in ATMs.

Additionally, the RNA-seq of liver suggested that esculetin induced a transcriptional alteration of hepatic genes (Figure S5A), and C/EBPβ-target genes were significantly enriched in esculetin-induced differentially expressed genes in livers (Figure S5B). The expressions of C/EBPβ and C/EBP were significantly increased after esculetin addition in HFD-fed mice (Figure S5C). Thus, the results indicated that esculetin could activate C/EBPβ *in vivo*.

## Discussion

In this study, we discovered that esculetin enhances lipid uptake through CD36-mediated phagocytosis in ATMs following high-fat meal intake. This leads to an increase in postprandial HDL cholesterol levels, promoting lipid circulation. Esculetin also augments bile acid content in bile, expediting the clearance of postprandial lipids. Mechanistically, esculetin directly interacts with and boosts the transcriptional activity of C/EBPβ, that positively regulates its target genes *Cd36* and downstream phagocytosis in macrophages and controls metabolic pathways associated with bile acids. In summary, esculetin promotes reverse cholesterol transport and excretion, potentially offering beneficial effects for accelerating postprandial cholesterol circulation and alleviating postprandial dyslipidemia.

Macrophage-mediated lipid uptake represents a fundamental physiological process that occurs across various tissues, including adipose tissue, liver, and lungs 19. Macrophages deficient in C/EBPβ show defects in the clearance of surfactant lipoproteins within the lung alveoli, resulting in pulmonary alveolar proteinosis like syndrome 57. However, under pathological conditions associated with metabolic disorders such as atherosclerosis, non-alcoholic fatty liver disease and obesity, the rate of lipid uptake by macrophages often exceeds their lipid processing capacity, leading to the formation of lipid-laden or foamy macrophages that may contribute to disease progression 58-60. While foam cell formation is generally considered a pathophysiological hallmark of disease states, macrophage lipid storage can also serve a protective function by mitigating the lipotoxic effects of lipid-rich environments and associated excessive lipid uptake 19. Notably, in obese adipose tissue, macrophages expressing Trem2 and associated genes involved in phagocytosis and lipid catabolism play a crucial protective role. Trem2 signaling drives the formation of lipid-associated macrophages within crown-like structures in adipose tissue, preventing adipocyte hypertrophy and maintaining systemic lipid homeostasis under obese conditions 58. This Trem2-dependent macrophage population exhibits conserved protective functions across multiple organs in various lipid disorder conditions. In the context of postprandial lipid metabolism, esculetin-mediated upregulation of CD36 expression may enhance lipid uptake and potentially promote foam cell formation. However, this process likely serves a protective role by accelerating lipid clearance and reducing the lipotoxic burden in lipid-rich environments. This mechanism represents an adaptive response that contributes to overall metabolic homeostasis and tissue protection. In our study, we specifically focus on the role of CD36 regulation by esculetin in managing excessive postprandial lipids under normal physiological conditions. We emphasize that the beneficial effects of esculetin are context-dependent, and further long-term studies are needed to fully understand its impact on lipid metabolism.

The macrophage liver X receptor α (LXRα) encoded by *Nr1h3* is a key transcription factor which controls the transcriptional regulation of lipid metabolism-associated genes involved in cholesterol uptake, efflux, transport, and excretion in multiple tissues. However, ATMs do not express LXRα 18,55,61; the key regulator of postprandial cholesterol circulation in ATMs is therefore still not well understood. Although this work lacks* in vivo* studies to confirm esculetin-induced elevation of HDL is due to the activation of C/EBPβ in ATMs, since the specific depletion of C/EBPβ in resident ATMs is technically difficult, our data suggested that C/EBPβ is expressed in ATMs and C/EBPβ controlled the uptake of lipid by macrophages, which is involved in the regulation of lipid metabolism and HDL generation.

Previous reports also suggested that C/EBPβ was expressed in myeloid cells, regulates the activation and/or differentiation of macrophages 62,63, and regulates lipid homeostasis during atherogenesis 64. Additionally, the direct binding of esculetin to C/EBPβ and the functional role of C/EBPβ in esculetin-induced uptake of lipid were confirmed, indicating that activation of C/EBPβ could enhance the uptake of lipid and lipid metabolism. Further, esculetin increased the accessibility of TSS regions on *Cd36*, *Cebpb* and *Nceh1*, binding by C/EBPβ, and esculetin-induced accessible chromatin alteration was highly enriched in the motif of C/EBPβ, suggesting that esculetin-induced dynamic epigenetic changes were controlled by C/EBPβ. Consistently, C/EBPβ was reported to regulate the lipid-induced epigenomic changes in macrophages including binding to *Cd36* and *PPAP2B* 54. Thus, we think that the expression of C/EBPβ in ATMs could be a key target transcription factor of esculetin controlling gene expression of lipid metabolism, especially the uptake of lipid, an initial stage of postprandial lipid circulation.

CD36 plays roles in regulating lipid metabolism, including regulation of dietary lipid clearance 23, maintenance of lipid utilization, lipid storage and lipolysis 65, and control of pro- and anti-inflammatory effects 66,67. Deficiency of CD36 could impair the clearance of lipoprotein lipase-mediated triglyceride 68, chylomicrons 21 and oxidized LDL 69, indicating the importance of CD36 in controlling the clearance of dietary lipid intake and reducing the risk of atherosclerosis. Furthermore, although the expression of CD36 was observed in various cell types and various tissues 70,71, the roles of CD36 differ according to different tissues. While conditional knockout models of CD36 could provide additional mechanistic insights, the broad expression of CD36 across multiple cell types and tissues poses a significant challenge in interpreting tissue-specific effects. CD36 is not only expressed in adipose tissue macrophages but also in other macrophages types involved in lipid metabolism 66. Therefore, even a conditional knockout approach may not fully isolate the contribution of ATMs CD36 to esculetin-regulated postprandial lipid metabolism. Our use of SSO provides a more targeted approach to validate CD36's necessity in this context. Future studies could explore advanced genetic tools or cell-type-specific delivery systems to further dissect the tissue-specific roles of CD36 in lipid metabolism. Esculetin was found to induce an increase in CD36^+^ macrophage subsets specifically in adipose tissue but not in the liver, and esculetin elevates HDL level in an eWAT dependent manner. Similarly, a high-fat meal has been shown to elevate CD36^+^ Tim4^+^ ATMs, which contribute to increased postprandial HDL levels but not Tim4^+^ liver Kupffer cells and peritoneal cavity macrophages 18 Adipose tissue adapts to food intake and hydrolyzes chylomicrons to release large amounts of lipid, which might suggest that ATMs specialize in the initiation of HDL-mediated RCT.

Pitavastatin has been demonstrated to downregulate CD36 expression through PPARγ inhibition in oxLDL-treated macrophage models 72. However, existing studies indicate that pitavastatin primarily enhances HDL levels by targeting apolipoprotein A-I (apoA-I) and ABCA1 in hepatocytes and intestinal cells, as well as by reducing endothelial lipase expression 73,74. Importantly, unlike the postprandial hyperlipidemia scenario described in our study, pitavastatin's HDL-elevating effects typically manifest after 4-6 weeks of treatment in dyslipidemic conditions 75. While both pitavastatin and esculetin demonstrated HDL-elevating effects in our study, it is crucial to emphasize that their mechanisms of action are fundamentally distinct. Notably, there is no established literature linking pitavastatin's CD36 downregulation to HDL production. In contrast, esculetin enhances CD36 expression in adipose tissue macrophages, thereby accelerating postprandial lipid metabolism and HDL generation. This mechanistic difference highlights that the effects of pitavastatin and esculetin on CD36 are involved in distinct biological processes. These results strongly support our conclusion that esculetin's HDL-elevating mechanism via CD36 operates through a pathway that is entirely separate from that of pitavastatin. Thus, our data suggested that CD36-mediated phagocytosis of ATMs is beneficial for cholesterol circulation after a high fat meal intake under physiological conditions. By promoting CD36-mediated uptake of lipid in ATMs, esculetin may exert benefits for maintaining postprandial homeostasis and postprandial dyslipidemia.

Role of HDL cholesterol as a substrate pool for bile acid precursors, does not directly drive the conversion of cholesterol to bile acids. The rate-limiting enzymatic conversion mediated by hepatic CYP7A1/CYP7B1 52,76. Thus, esculetin may simultaneously engage eWAT-mediated HDL biogenesis and hepatic-intrinsic pathways to optimize postprandial lipid metabolism; while eWAT-derived HDL expands the mobilizable cholesterol pool, the liver autonomously regulates its conversion to bile acids based on metabolic demand. This compartmentalized regulation explains why bile acid accumulation remains intact despite eWAT removal. Therefore, our findings suggest that increased HDL produced by ATMs may provide additional substrate for bile acid synthesis, thereby promoting postprandial lipid circulation.

## Conclusions

This study suggests that esculetin may elevate postprandial HDL levels and accelerate postprandial cholesterol circulation and excretion, potentially through increased CD36-mediated phagocytosis of ATMs and activation of C/EBPβ.

## Supplementary Material

Supplementary figures and table.

## Abbreviations

APTES: (3-aminopropyl) triethoxysilane
ATAC: Chromatin accessibility profiling via assay for transposase-accessible chromatin
ATMs: Adipose tissue macrophages
C/EBPβ: CCAAT enhancer-binding protein beta
CD: control diet
ChIP-seq: chromatin Immunoprecipitation sequencing
DEGs: significantly differentially expressed genes
DEMs: significantly differentially expressed metabolites
ESI: Electrospray ionization
eWAT: Epididymal white adipose tissue
GSEA: gene set enrichment analysis
H&E: hematoxylin and eosin
HDL: High-density lipoprotein
HFD: high-fat diet
HMG-CoA: Hydroxymethylglutaryl coenzyme A
HRP: horseradish peroxidase
IGV: Integrative Genomics Viewer
IHC: Immunohistochemical
KEGG: Kyoto Encyclopedia of Genes and Genomes
LDL: Low-density lipoprotein
MCE: Macrophage cholesterol efflux
MSEA: metabolic set enrichment analysis
OPLS-DA: orthogonal projection to latent structures-discriminant analysis
PLS-DA: partial least squares discriminant analysis
PVDF: polyvinylidene fluoride
qRT-PCR: Real-Time Reverse Transcription PCR
RCT: Reverse cholesterol transport
RPKM: reads per kilobase per million
SDS-PAGE: sodium dodecyl sulfate-polyacrylamide gel electrophoresis
SMPDB: Small Molecule Pathway Database
SSO: Sulfo-N-succinimidyl oleate
TSS: transcription start site
UHPLC-MS/MS: liquid chromatography-tandem mass spectrometry
VIP: Variable importance in the projection
