# Supplementary material for: A small molecule esculetin accelerates postprandial lipid clearance involving activation of C/EBPβ and CD36-mediated phagocytosis by adipose tissue macrophages
Source: Theranostics. 2025 Apr 28;15(12):5910–30. doi: 10.7150/thno.110207 (PMC12068311; doi:10.7150/thno.110207)
Supplement: Supplementary file 1 — Supplementary figures and table. [file thnov15p5910s1.pdf]

Supporting Information

Figure S1

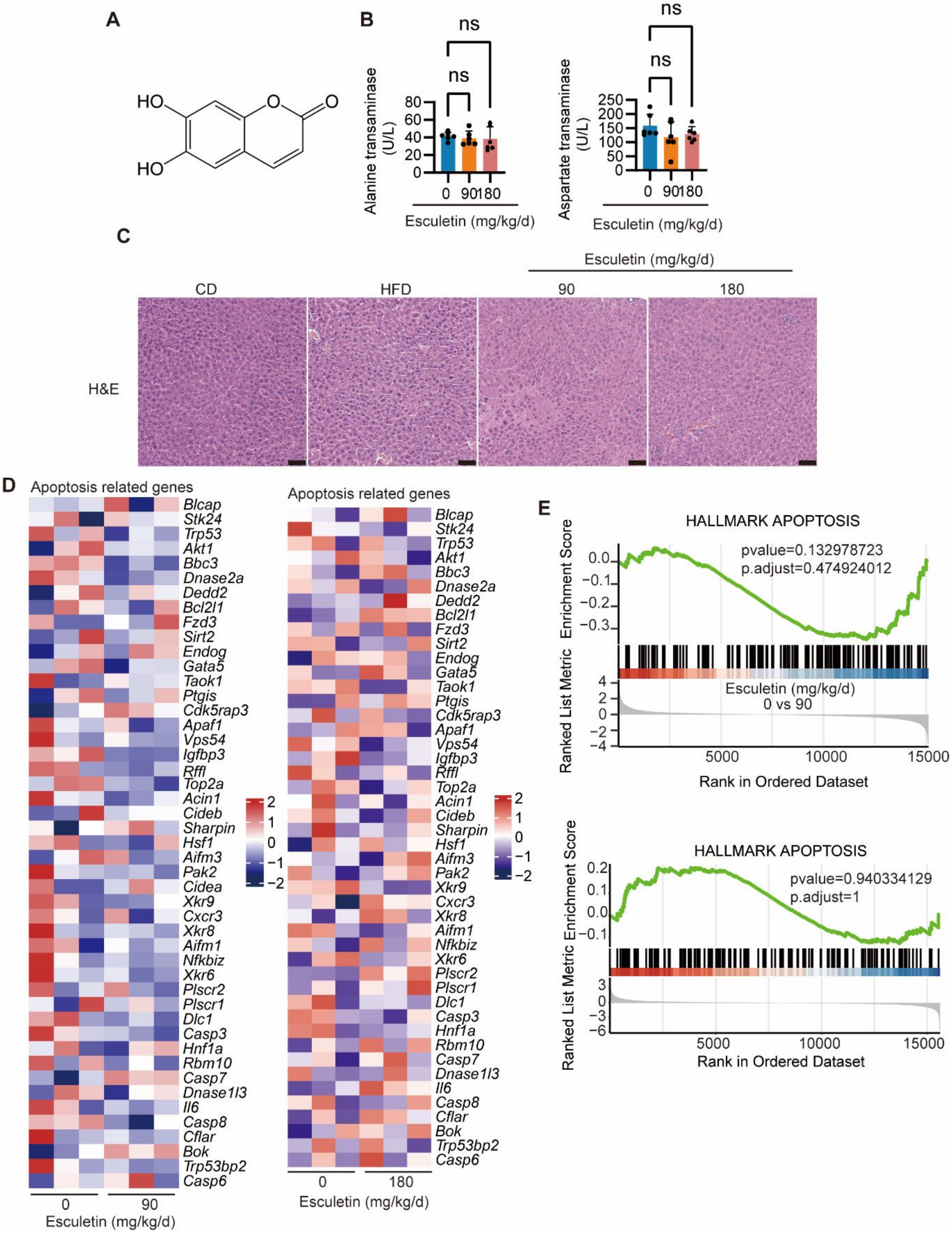

Figure S1. Esculetin did not exert hepatic toxicity at the doses of 90 and 180 mg/kg/d. (A) The structure of esculetin. (**B**) The serum levels of liver enzymes alanine aminotransferase (ALT) and aspartate transaminase (AST) in HFD-fed mice with or without esculetin at indicated doses. Data points in graphs show individual mice (n = 7) over 2 experiments, analyzed by one-way ANOVA with Tukey's multiple comparisons test for ALT or Kruskal-Wallis with Dunn's multiple comparisons test for AST. (**C**) Hematoxylin and eosin (H&E) staining of hepatic tissue. (**D**) The heatmap of apoptosis-related genes from the liver. RNA sequencing was performed using the livers from HFD-fed mice with 0, 90, 180 mg/kg/d esculetin treatment (n = 3). One sample contains two mice. (**E**) Gene set enrichment analysis (GSEA) against apoptosis pathway is shown using the RNA sequencing data from the livers.

Figure S2

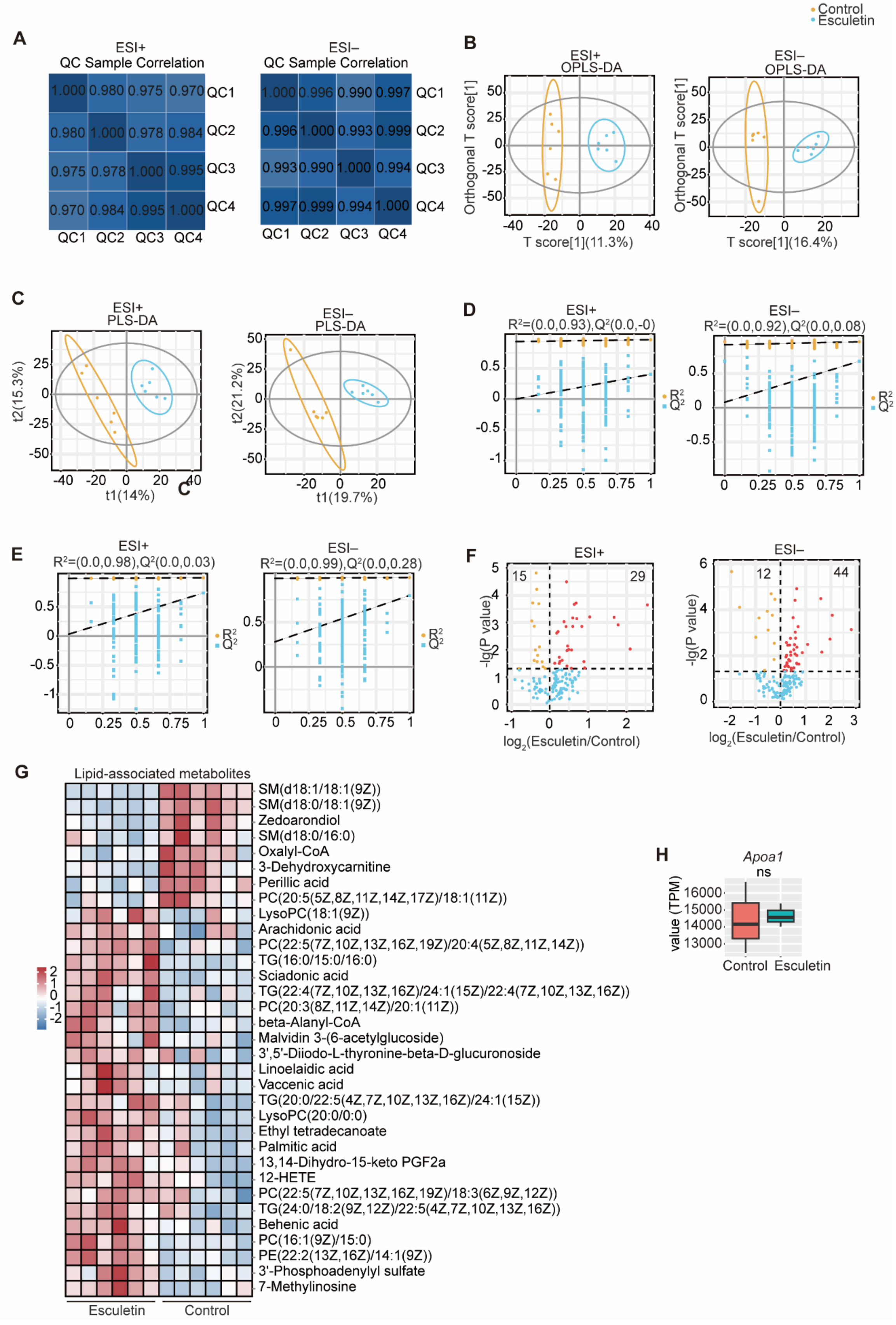

Control
Esculetin
A
ESI+
QC Sample Correlation
1.000 0.980 0.975 0.970 QC1
0.980 1.000 0.978 0.984 QC2
0.975 0.978 1.000 0.995 QC3
0.970 0.984 0.995 1.000 QC4
QC1 QC2 QC3 QC4
ESI–
QC Sample Correlation
1.000 0.996 0.990 0.997 QC1
0.996 1.000 0.993 0.999 QC2
0.993 0.990 1.000 0.994 QC3
0.997 0.999 0.994 1.000 QC4
QC1 QC2 QC3 QC4
B
ESI+
OPLS-DA
Orthogonal T score[1]
T score[1](11.3%)
ESI–
OPLS-DA
Orthogonal T score[1]
T score[1](16.4%)
C
ESI+
PLS-DA
t2(15.3%)
t1(14%)
ESI–
PLS-DA
t2(21.2%)
t1(19.7%)
D
ESI+
R²=(0.0,0.93),Q²(0.0,-0)
ESI–
R²=(0.0,0.92),Q²(0.0,0.08)
R²
Q²
E
ESI+
R²=(0.0,0.98),Q²(0.0,0.03)
ESI–
R²=(0.0,0.99),Q²(0.0,0.28)
R²
Q²
F
ESI+
15
29
-lg(P value)
log2(Esculetin/Control)
ESI–
12
44
-lg(P value)
log2(Esculetin/Control)
G
Lipid-associated metabolites
SM(d18:1/18:1(9Z))
SM(d18:0/18:1(9Z))
Zedoarondiol
SM(d18:0/16:0)
Oxalyl-CoA
3-Dehydroxycarnitine
Perillic acid
PC(20:5(5Z,8Z,11Z,14Z,17Z)/18:1(11Z))
LysoPC(18:1(9Z))
Arachidonic acid
PC(22:5(7Z,10Z,13Z,16Z,19Z)/20:4(5Z,8Z,11Z,14Z))
TG(16:0/15:0/16:0)
Sciadonic acid
TG(22:4(7Z,10Z,13Z,16Z)/24:1(15Z)/22:4(7Z,10Z,13Z,16Z))
PC(20:3(8Z,11Z,14Z)/20:1(11Z))
beta-Alanyl-CoA
Malvidin 3-(6-acetylglucoside)
3',5'-Diiodo-L-thyronine-beta-D-glucuronoside
Linoelaidic acid
Vaccenic acid
TG(20:0/22:5(4Z,7Z,10Z,13Z,16Z)/24:1(15Z))
LysoPC(20:0/0:0)
Ethyl tetradecanoate
Palmitic acid
13,14-Dihydro-15-keto PGF2a
12-HETE
PC(22:5(7Z,10Z,13Z,16Z,19Z)/18:3(6Z,9Z,12Z))
TG(24:0/18:2(9Z,12Z)/22:5(4Z,7Z,10Z,13Z,16Z))
Behenic acid
PC(16:1(9Z)/15:0)
PE(22:2(13Z,16Z)/14:1(9Z))
3'-Phosphoadenylyl sulfate
7-Methylinosine
Esculetin
Control
H
Apoa1
ns
value (TPM)
16000
15000
14000
13000
Control
Esculetin

Figure S2. The multivariate statistical analysis of serum metabolites. (**A**) The *Pearson* correlation coefficients among the quality control (QC) samples. (**B**) Orthogonal projection to latent structures-discriminant analysis (OPLS-DA) score graphs. (**C**) Partial least squares discriminant analysis (PLS-DA) score graphs. Permutation test results of the OPLS-DA (**D**) and PLS-DA (**E**) model in the positive and negative ion modes. (**F**) The volcano plot of esculetin-induced significantly differentially expressed metabolites (DEMs) identified from positive and negative ion modes (n = 6). (**G**) Heatmap of lipid-associated metabolites. **(H)** Transcription levels of *Apoa1*, expressed as transcripts per kilobase million (TPM), were quantified by RNA-seq analysis of liver samples from high-fat diet-fed mice treated with a control or 90 mg/kg/day esculetin (n = 3).

Figure S3

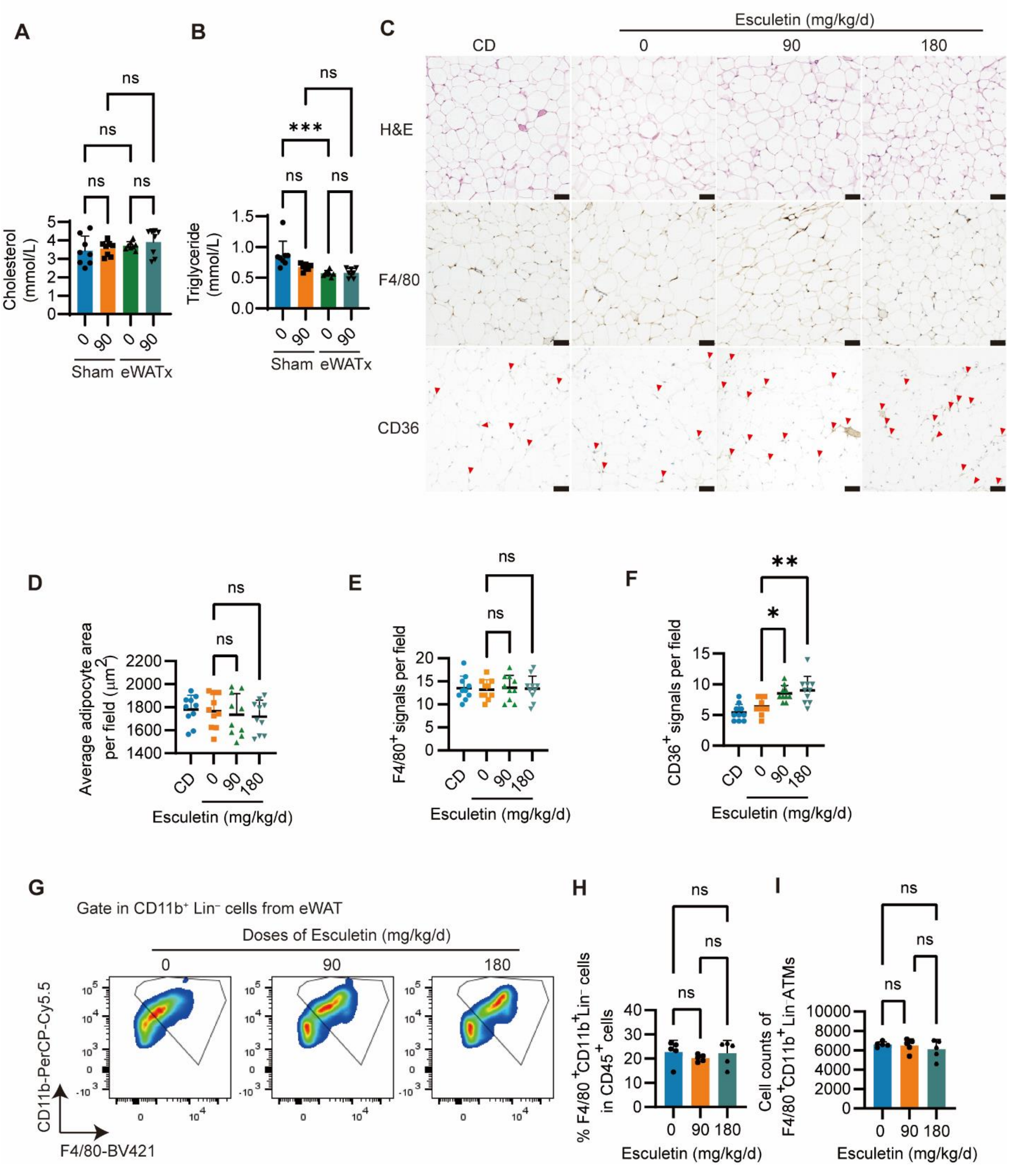

Figure S3. Quantitative and histological analysis of lipid metabolism and adipose tissue macrophage in eWAT with Esculetin treatment. (A, B) Quantification of postprandial serum Cholesterol and Triglycerides. **(C)** Hematoxylin and eosin (H&E) staining and

immunohistochemistry (IHC) of F4/80 and CD36 in eWATs. Scale bar, 50 μm. **(D)** The average adipocyte area per field was quantified. **(E)** F4/80$^+$ cells per field were quantified. **(F)** CD36$^+$ cells per field were quantified. Arrow indicated the positive signals. Data points represent individual fields (n = 9) from 3 mice across two independent experiments. (**G**) Representative flow cytometry plots of F4/80$^+$ CD11b$^+$ macrophage gating in CD11b$^+$ Lineage$^-$ cells from epididymal white adipose tissue (eWAT). The lineage markers used include CD19, TCRβ, Ly-6G, and Ly-6C. (**H**) Percentage of F4/80$^+$ CD11b$^+$ Lineage$^-$ macrophages among CD45$^+$ cells in eWAT. (**I**) Absolute counts of F4/80$^+$ CD11b$^+$ macrophages per mouse. Statistical analysis was performed using one-way ANOVA with Tukey's multiple comparisons test (A, D-F, H-I), and the Kruskal-Wallis test with Dunn's multiple comparisons test (B).

Figure S4

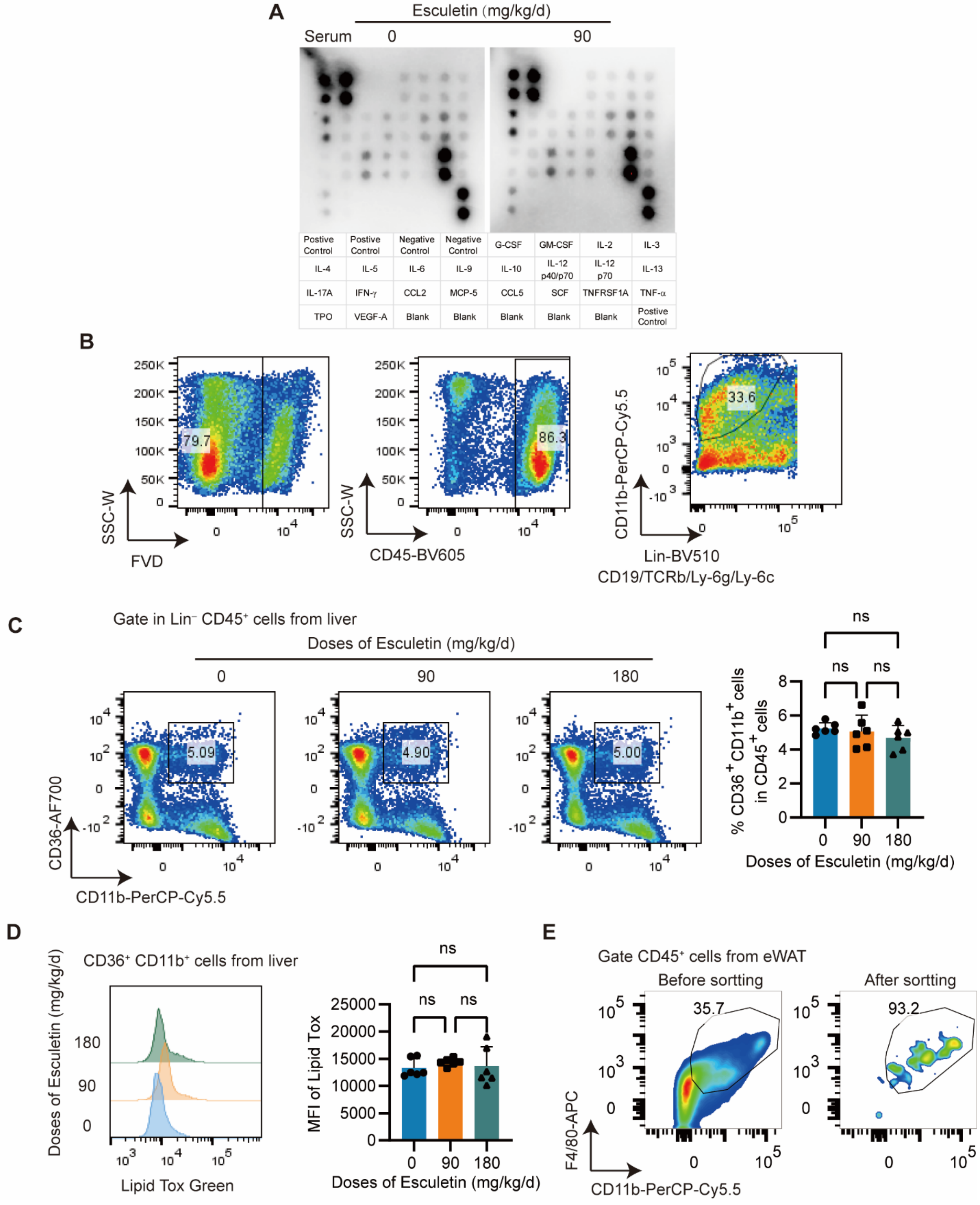

Figure S4. Esculetin did not change the macrophages subsets from liver. (A) Representative image of cytokine/chemokine antibody array using serum from HFD-fed mice treated with either

0 or 90 mg/kg/day of esculetin. One representative out of two similar experiments is displayed. One sample contains three mice. (**B**) Representative gating strategies of flow cytometry analysis. (**C**) The percentages of $CD36^+$ $CD11b^+$ cells in $CD45^+$ cells from liver. Data points represent individual mice (n = 6) over two experiments, analyzed by ANOVA with Tukey's multiple comparisons test. (**D**) Mean fluorescence intensity (MFI) of LipidTOX in $CD36^+$ $CD11b^+$ cells from liver. Data points represent individual mice (n = 6) over two experiments, analyzed by Kruskal-Wallis with Dunn's multiple comparisons test. **(E)** Representative flow cytometry of ATMs before and after sorting.

Figure S5

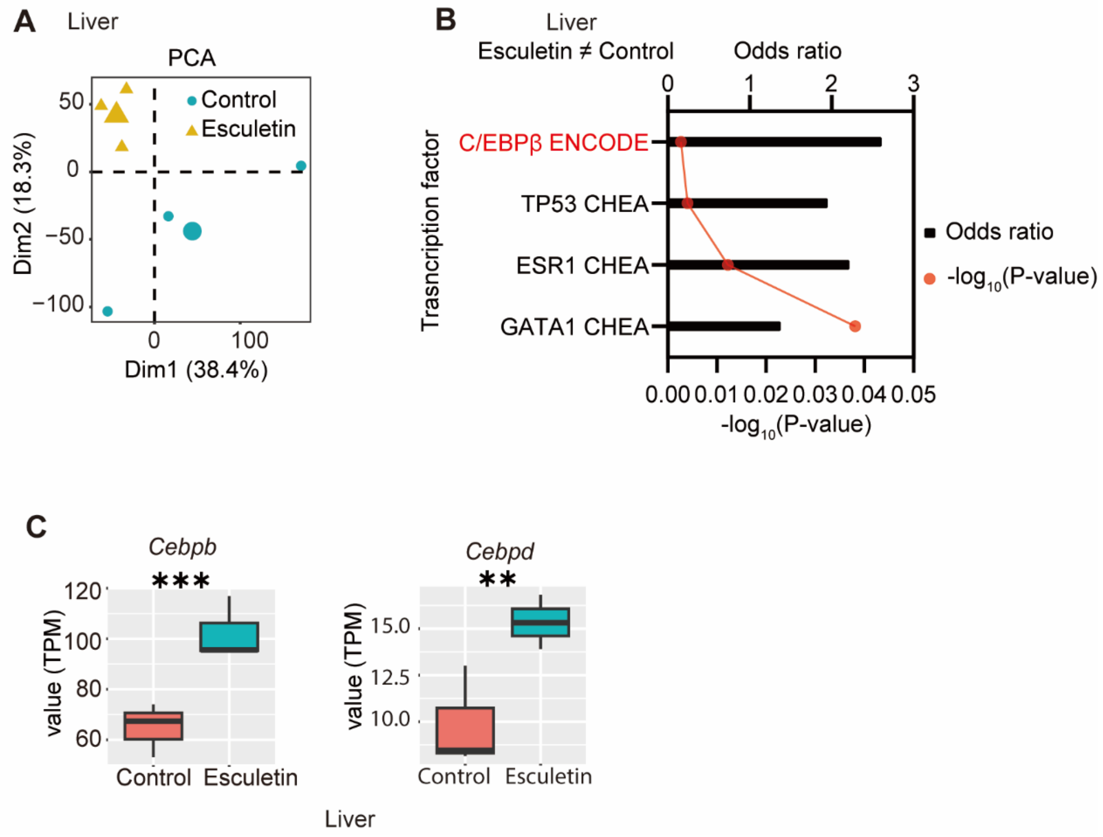

Figure S5. C/EBPβ-target genes were significantly enriched in esculetin-induced differentially expressed genes in livers. RNA sequencing was performed using the livers from HFD-fed mice with or without 90 mg/kg/d esculetin treatment (n = 3). One sample contains two mice. **(A)** Principal component analysis plot is shown. **(B)** Esculetin-induced significantly differentially expressed genes (DEGs) were used for enrichment analysis against TRRUST transcription factor gene sets. **(C)** The expression levels of *Cebpb* and *Cebpd* in livers were quantified by RNA-seq.

Figure S6

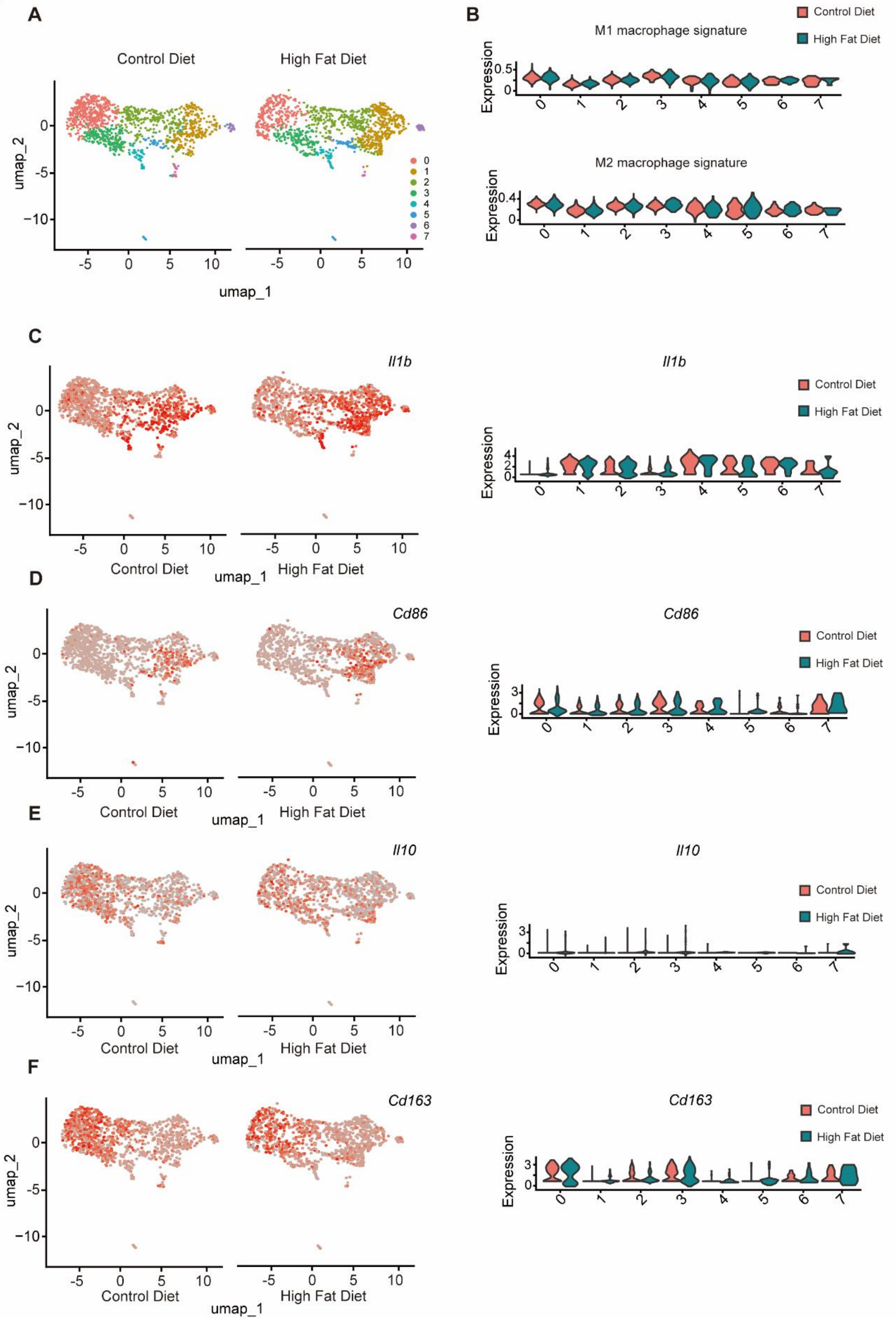
A
Control Diet
High Fat Diet
umap_2
umap_1
0
1
2
3
4
5
6
7
B
M1 macrophage signature
M2 macrophage signature
Control Diet
High Fat Diet
Expression
C
Il1b
D
Cd86
E
Il10
F
Cd163

Figure S6. Expression profiles of M1/M2 macrophage markers in postprandial high-fat diet-induced macrophages. (A) Unsupervised clustering of ATMs using UMAP, where each dot represents a single cell, colored by cluster assignment, with separate groupings for control diet and high-fat diet conditions. **(B)** Violin plots showing the expression patterns of M1/M2 macrophage signature gene sets across different clusters and groups. **(C-F)** Dot plots and violin plots showing the cluster and group-specific expression profile of *Il1b*, *Cd86*, *Il10*, and *Cd163*, respectively.

Figure S7

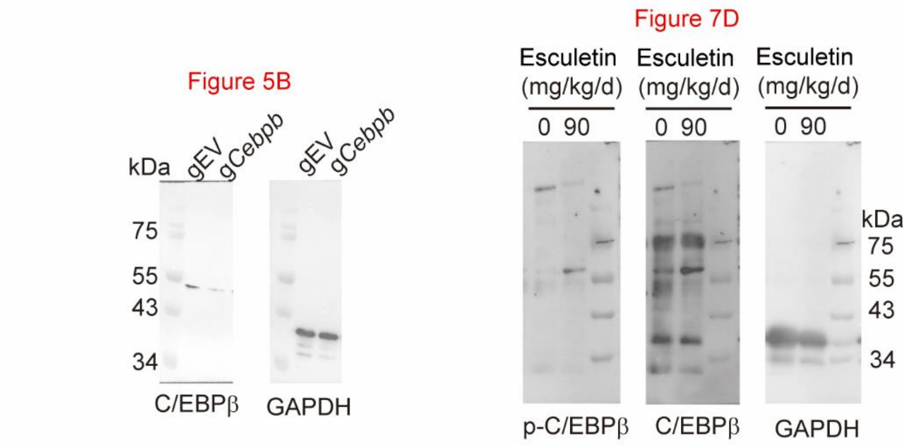

Figure S7. Whole gel images of Western blot analysis.

Supplementary Tables

Table S1. Key resources table

Reagent or resource	Identifier	Source
**Antibodies**		
Brilliant Violet 605 anti-mouse CD45 Antibody	103155	Biolegend
Brilliant Violet 510 anti-mouse CD19 Antibody	115545	Biolegend
Brilliant Violet 510 anti-mouse TCR β chain Antibody	109233	Biolegend
Brilliant Violet 510 anti-mouse Ly-6G Antibody	127633	Biolegend
Brilliant Violet 510 anti-mouse Ly-6G/Ly-6C (Gr-1) Antibody	108457	Biolegend
PerCP/Cyanine5.5 anti-mouse/human CD11b Antibody	101228	Biolegend
Alexa Fluor 700 CD36 Monoclonal Antibody (HM36)	56-0362-82	Thermo Fisher
Brilliant Violet 421 anti-mouse F4/80 Antibody	123131	Biolegend
APC anti-mouse Tim-4 Antibody	130022	Biolegend
C/EBPB Rabbit pAb	A0711	Abclonal
Phospho-C/EBPB-T235 Rabbit pAb	AP1055	Abclonal
Anti -F4/80 Rabbit pAb	GB113373	Servicebio
GAPDH Mouse mAb	AC033	Abclonal
CD36 antibody	sc-7309	Santa Cruz
Goat anti-Rabbit IgG Secondary Antibody, HRP	GB23303	Servicebio
Goat anti-Rabbit IgG Secondary Antibody, Alexa Fluor 488	A-11008	Thermo Fisher
C/EBPB antibody (H-7) for ChIP	sc-7962	SCBT
Mouse IgG antibody	sc-52336	SCBT
**Chemicals, kit and buffer**		
Esculetin	D77970	ACMEC
Simvastatin	H20084420	Xinqi

Pitavastatin	H20193061	Salubris
Tween-80	T8360	Solarbio
Sulfo-N-succinimidyl oleate	HY-112847A	MedChemExpress
Puromycin dihydrochloride	HY-B1743A	MedChemExpress
Dimethyl sulfoxide	D8371	Solarbio
Methanol (LC-MS grade)	CAEQ-4-000306-4000	Anpel
Acetonitrile (LC-MS grade)	CAEQ-4-000308-4000	Anpel
Ammonium ethanoate (LC-MS grade)	73594	Sigma
Ammonium hydroxide (LC-MS grade)	60-046-886	Fisher Scientific
Dulbecco's Modified Eagle Medium	PM150210	Procell
Fetal Bovine Serum	164210-50	Procell
Penicillin and streptomycin	PB180120	Procell
DIL-ox-LDL	YB-0010	Yiyuan Biotechnologies
Collagenase D	11088866001	Roche
Liberase	5401127001	Roche
Dnase I	D5025	Lablead
Percoll	17089101	Cytiva
Prestained Protein marker	P1018	Lablead
WesternBright ECL HRP substrate	K-12045-D50	Advansta
FcR blocking reagent	130-092-575	Miltenyibiotec
Fixable Viability Dye eFluor 780	65-0865-14	Thermo Fisher
RPMI 1640	L210KJ	Basalmedia Technologies
Fetal bovine serum for flow cytometry	C4055L1050	Life-ilab
eBioscience Foxp3 / Transcription Factor Staining Buffer Set	00-5523-00	Invitrogen
IC fixation buffer	00-8222-49	Invitrogen
Lipid Tox Green Neutral Lipid Stain	H34475	Thermo Fisher
Trizol	15596026	Thermo Fisher
Nebnext Ultra RNA Library Prep Kit for Illumina	E7530	New England Biolabs
TruePrep DNA Library Prep kit V2 for Illumina	TD501	Vazyme
Qiagen PCR purification kit	28104	Qiagen
NEBNext Ultra II Q5 Master Mix	M0544	New England Biolabs

NovoNGS Index Kit for Illumina	N239	novoprotein
Qiagen MiniElute Reaction Celanup Kit	28206	Qiagen
RNA-direct SYBR Green Real time PCR Master Mix	QRT-201 100	TOYOBO
DAB staining kit	G1212	Servicebio
H&E staining kit	G1076	Servicebio
Human C/EBPβ recombinant protein	P17676	Raybiotech
Silicon-on-Sapphire sensing plate (11-02, R Plane)	FmSOS1010046S2FT05US	MTI Corporation
(3-aminopropyl) triethoxysilane	440140	Sigma-Aldrich
bis(sulfosuccinimidyl)suberate	S5799	Sigma-Aldrich
Pierce 16% Formaldehyde (w/v), Methanol-free	28908	Thermo Fisher
Dynabeads Protein G for Immunoprecipitation	10003D	Thermo Fisher
Cytokine Array C1 kit	AAM-CYT-1-8	Raybiotech
Hieff qPCR SYBR Green Master Mix	11204ES08	Yeansen
**Software and Algorithms**		
Xcalibur (v4.4)		Thermo Fisher
Proteowizard (v3.0)		
R (v4.2.1)		
XCMS package (v3.22.0)		
Biotreedb MS2 database (v2)		Gene Denovo Biotechnology
Ropls package (v1.32.0)		
Mesap package (v0.99.0)		
HISAT (v2.2.1)		
Featurecounts (v2.0.1)		
Limma v3.56.1		
Gseabase (v1.62.0)		
Clusterprofiler (v4.8.1)		
Trim-Galore (version 0.6.10)		
Bowtie2 (version 2.5.1)		
MACS2 (version 2.2.6)		

Sambamba (version 1.01)		
HOMER (version 4.10.4)		
Integrative Genomics Viewer		
DeepTools (version 3.4.1)		
DESeq2		
Flow Jo (v.10.0.7)		Becton Dickinson
Prism 9		Graphpad
Seurat R package (v2.6)		
**Mice and Others**		
C57BL6J mice		Gempharmatech
RAW 264.7 cell	CL-0190	Procell
Lenticrispr v2 plasmid	52961	Addgene
Control diet	D12450J	Research Diets
High fat diet	D12492	Research Diets
**Oligos**		
**Primers**	**Fw**	**Rv**
Cd36	GGACATTGAGATTCTTTTCCTCTG	GCAAAGGCATTGGCTGGAAGAAC
NcheI	CGGTATTTCTGGAGACAGTGCTG	GGTGTGTTGAAGTCCAAAGCCTG
Cebpb	CAACCTGGAGACGCAGCACAAG	GCTTGAACAAGTTCCGCAGGGT
Gapdh	CATCACTGCCACCCAGAAGACTG	ATGCCAGTGAGCTTCCCGTTCAG
*Cd36*_ChIP		
P1	ACTTCCCAGATTCAGATGGAGC	AGGCAATCGCTCTAACAGGC
P2	ACCCCATGCTGCTCTGCTAT	ACAGAGCATTGGGAGTTCCTC
P3	AGCATATTGGGTGCTGACCA	CCAGGTAATCCACACTCCGT
**gRNA**		
Cebpb	GCTGCTTGAACAAGTTCCGC	
